# Modulating Placebo Effects in Clinical Trials: Study Team Briefing to Optimize Drug–Placebo Differences

**DOI:** 10.1111/cts.70399

**Published:** 2025-11-13

**Authors:** Martin Coenen, Ulrike Bingel, Maria Soledad Berdaguer, Christine Fuhrmann, Robert Németh, Jens Rengelshausen, Gunther Hartmann, Christoph Coch, Manfred Schedlowski

**Affiliations:** ^1^ Clinical Study Core Unit, Study Center Bonn (SZB) University Hospital Bonn Bonn Germany; ^2^ Institute of Clinical Chemistry and Clinical Pharmacology University Hospital Bonn Bonn Germany; ^3^ Department of Neurology, Center for Translational Neuro‐ and Behavioural Sciences University Hospital Essen Essen Germany; ^4^ Institute of Medical Biometrics, Informatics and Epidemiology University Hospital Bonn Bonn Germany; ^5^ Institute for Occupational, Social and Environmental Medicine Uniklinik RWTH Aachen Aachen Germany; ^6^ Clinic and Policlinic for Neurosurgery Leipzig Germany; ^7^ Institute of Medical Psychology and Behavioral Immunobiology, Center for Translational Neuro‐ and Behavioural Sciences University Hospital Essen Essen Germany; ^8^ Department of Clinical Neuroscience, Osher Center for Integrative Medicine Karolinska Institutet Stockholm Sweden

**Keywords:** analgesic therapy, placebo effect, placebo response, study team

## Abstract

Clinical trials often face challenges with placebo effects that can mask actual drug effects. We evaluated whether briefing the study team members on placebo mechanisms influenced analgesia. In this study, we compared the analgesic effects of oxycodone and placebo in three groups of 32 subjects. The groups were treated by an untrained study team (*Arm A*), a team trained to maximize (*Arm B*) and a team trained to minimize placebo effects (*Arm C*). The primary outcome was the reduction of pain during the cold pressor test, assessed by the area under the pain curve of the visual analog scale before and after blinded administration of oxycodone or placebo. Oxycodone and placebo demonstrated the expected differences in pain reduction across all study arms. Briefing the study team did not significantly affect pain reduction or treatment expectation, regardless of treatment. However, treatment expectations were more pronounced with oxycodone compared to placebo, showing a positive correlation of expectation and treatment effect only in the oxycodone group, possibly reflecting the influence of unblinding due to adverse effects. These findings suggest that a brief training of the study team may not be sufficient to alter treatment expectations and placebo analgesia. This insight will inform future efforts to apply placebo research to optimize blinded trial design and drug treatments in clinical settings.

**Trial Registration:** DRKS 00013586 (German Clinical Trials Register), registered on December, 22nd 2017; URL: https://www.drks.de/drks_web/setLocale_EN.do

**Abbreviations**
AUC: area under the curve
BMQ: beliefs about medicines questionnaire
CPT: cold pressor test
GASE: Generic Assessment of Side Effects
HADS‐D: Hospital Anxiety and Depression Scale—German Version
HPA: hypothalamus‐pituitary–adrenal
ITT: intention‐to‐treat
SSAS‐D: Somatosensoric Amplification Scale
STADI: State–Trait‐Angst‐Depressions‐Inventar
STD: standard deviation
STE: standard error
TICS: Trierer Inventar zum chronischen Stress
VAS: visual analogue scale

**Study Highlights**

- What is the current knowledge on the topic?
  ○ Placebo effects can obscure true drug effects in clinical trials, reducing assay sensitivity and complicating drug approval processes. While interactions between study staff and participants may influence placebo effects, the impact of targeted training for study teams remains unclear.
- What question did this study address?
  ○ This study evaluated whether briefing of a study team to maximize or minimize placebo effects influences analgesic outcomes and treatment expectations in a controlled clinical trial setting.
- What does this study add to our knowledge?
  ○ Briefing the study team had minimal effects on treatment expectations and no significant impact on pain reduction. However, the study identified a positive correlation between higher treatment expectations and enhanced analgesic effects under oxycodone, highlighting challenges associated with potential unblinding due to adverse effects and indicating interactive effects between treatment expectations, pharmacological effects and treatment outcomes.
- How might this change clinical pharmacology or translational science?
  ○ The findings emphasize the need for rigorous study designs to modify placebo and expectation effects in early‐phase clinical trials. Future research should explore more intensive and prolonged training interventions to better influence placebo and nocebo effects in clinical research.

## 1 Introduction

The placebo response in clinical trials refers to symptom improvement after administering an inert substance [1]. It encompasses various factors such as the natural history of a disease, fluctuation of symptoms, response bias, concurrent interventions, statistical phenomena like regression to the mean and an expectancy‐induced psychoneurobiological component, also referred to as the placebo effect [2, 3]. While placebos help to differentiate these non‐specific effects from a drug's specific effect in clinical trials [1], placebo effects can also obscure true drug effects, impairing assay sensitivity and potentially complicating the drug approval process [4]. This can jeopardize or, in the worst case, even prevent the approval of a new drug for marketing authorization despite its intrinsic pharmacological efficacy [5]. Thus, systematically reducing placebo effects in clinical research to optimize “assay sensitivity” and highlight genuine drug benefits is crucial for advancing drug development [3].

Over the past decades, research has identified patients' expectations as the key determinant of placebo effects. These in turn are driven by any prior information available to the individual to predict treatment outcome, such as verbal information, own prior experiences and the observation of treatment benefits in others [6]. Furthermore, contextual factors and the quality and quantity of practitioner‐patient interactions play a key role in shaping expectations and their effect on health outcome [7]. These effects are best demonstrated in the field of pain, where abundant evidence shows that the subject's or patient's expectations significantly affect both, subjective pain perception and neurobiological brain activity [8, 9, 10].

In clinical trials, especially phase I trials that often require a stay on a specialized ward, frequent interactions between the study team and participants are common. Evidence suggests that educating trial participants about placebo mechanisms can reduce placebo responses [11, 12], which frequently undermine trials [8, 13]. Therefore, briefing a study team on placebo effects and how they can be influenced could similarly affect outcomes in phase I trials.

To examine whether educating the study team on placebo effects affects the analgesia of study participants, we conducted the PINgPOng study—a prospective, randomized, double‐blind, placebo‐controlled clinical trial with a 2‐sequence, 2‐period cross‐over design. We enrolled three cohorts of 32 subjects each, treated by different study teams: (i) an untrained team (*Arm A*), (ii) a team trained to maximize placebo effects (*Arm B*), and (iii) a team trained to minimize placebo effects (*Arm C*). The team was unaware of the trial's actual objectives only in *Arm A*, while all participants remained blinded throughout the trial. We used the analgesic drug oxycodone in the cold pressor test (CPT), a standard model for studying (placebo) analgesia [14], since placebo effects play a relevant role in analgesic therapy and there are various proven analgesic mechanisms related to placebo reactions [15, 16]. To mirror a realistic and standardized early clinical trial environment, we opted for a simulated inpatient setting of a professional early clinical trial unit, maintaining close, controlled interactions over multiple days with healthy volunteers, as typical in early drug development.

## 2 Methods and Design

### 2.1 Study Design

**FIGURE 1:**
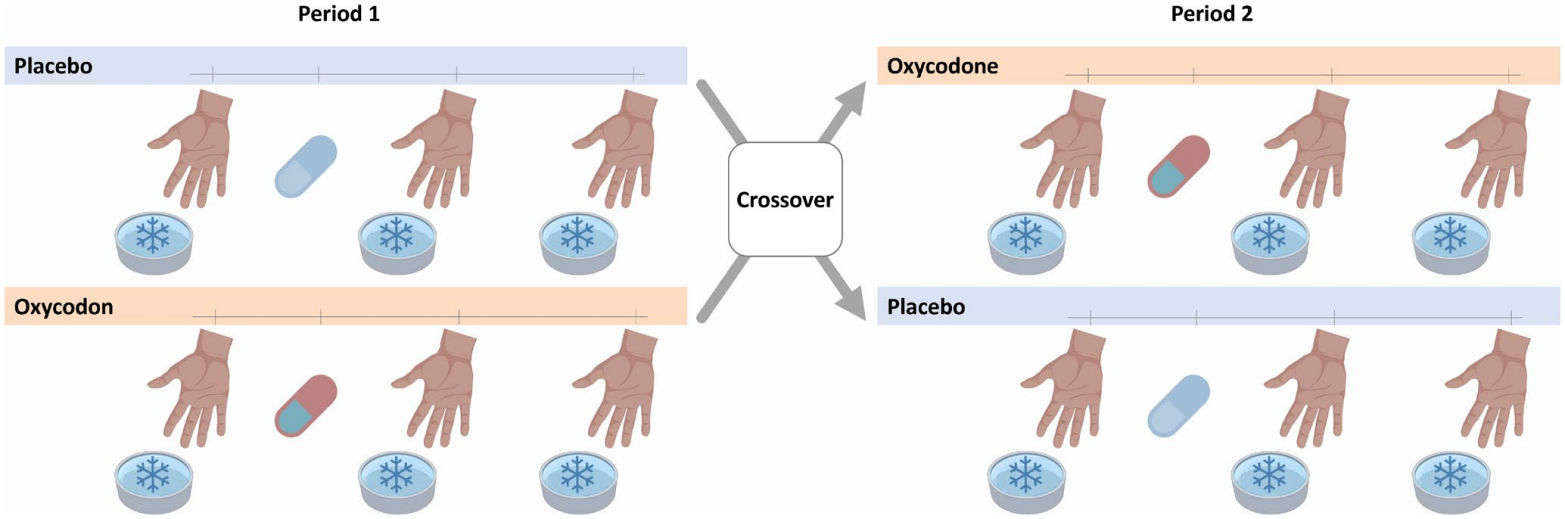
Study Design. On the treatment day the first cold pressor test (CPT) was conducted as a reference 1 h before the administration of 15 mg oxycodone or placebo. Two additional CPTs were performed one and 2 h later. Procedures were repeated for the second period of the crossover study between 7 and 21 days after the end of the first period. Three different cohorts were treated consecutively: (i) with a normally acting “untrained study team”, blinded to the actual study rationale, Arm A, “untrained”, (ii) with a study team trained how to maximize placebo effects (Arm B, “maximize”) after information about the true nature of the study, and (iii) with a study team trained how to minimize placebo effects (Arm C, “minimize”).

The PINgPOng study was designed as a prospective, randomized, double‐blind, single‐center, placebo‐controlled trial with a 3‐arm, 2‐sequence, 2‐period crossover design within each study arm (Figure 1). It was approved by the independent ethics committee of the University of Bonn (reference no: 278/17) and conducted in the Phase I Unit of the Institute of Clinical Chemistry and Clinical Pharmacology of the University Hospital Bonn in accordance with the principles of ICH‐GCP and the Declaration of Helsinki. The study was registered in the DRKS 00013586 (German Clinical Trials Register) on December 22nd, 2017.

The details of the study design and procedures have been previously published [17]. Briefly, the first cohort of the study (*Arm A, “untrained”*) was conducted with a normally acting “naïve study team”, which was blinded to the actual rationale of the study. Before the second cohort (*Arm B, “maximize”*) entered the study, the study team was informed about the true nature of the study and received an “educational short course” (see “briefing procedure”) on mechanisms steering placebo responses, with the intention of maximizing placebo effects in participants of study *Arm B*. Before the third cohort (*Arm C, “minimize”*) entered the study, the team was briefed on how to minimize placebo effects. The three different cohorts were treated consecutively. The study participants were not informed about the actual study goal or the nature of the different briefing sessions and stages of the study team. Instead, they were told that the study analyzed general aspects of pain perception.

For detailed information on inclusion and exclusion criteria of the study participants, study coordination and data management, please refer to the Supporting Information.

### 2.2 Participants

On the day before administration of the study drug, healthy female and male subjects aged between 18 and 60 years, determined through medical evaluation including medical history, physical examination, laboratory tests, and 12‐lead ECGs during the screening visit, were admitted to the study ward. A cold pressor test (CPT) was performed. Subjects with an area under the curve (AUC) of less than 10% were excluded from study participation to ensure that they showed a measurable response to the pain provocation in general. Subsequently, all other applicable inclusion and exclusion criteria for the three cohorts were assessed, and eligible subjects were randomized in a 1:1 ratio, stratified by sex, to one of the two blinded treatment sequences of the study medication (oxycodone—placebo or placebo—oxycodone).

### 2.3 Trial Investigations and Procedures

#### 2.3.1 Analgesic Treatment

The study medication was oxycodone, a semi‐synthetic opioid with analgesic effects on acute and chronic pain conditions, administered as a single dose of 15 mg. Its efficacy in the pain model used in this study has been validated in several studies on pain stimulation, demonstrating sufficient efficacy and suitability for placebo‐controlled studies [18, 19].

#### 2.3.2 Cold Pressor Test (CPT)

Interindividual and group‐specific differences in analgesic effect were assessed using the cold pressor test (CPT), an established and widely used experimental pain model for acute tonic pain and pain tolerance, sensitive to opioid analgesia [20, 21]. In the CPT, participants immersed their non‐dominant hand in 6°C cold water for as long as tolerable, up to a safety maximum of 120 s, while continuously rating pain intensity on a 100‐point visual analogue scale (VAS; assessments: “0: no pain”, “100: unbearable pain”) using a mechanical sliding lever. Participants could terminate the test at any time. The start and termination times (tolerance) of the CPT were logged by study staff using a button.

#### 2.3.3 Study Procedures

Participants were recruited via public ads and database searches between December 2017 and October 2020, signing informed consent at their screening visit. They underwent medical documentation, ECG, vital signs, lab tests, and psychometric evaluations (STADI, HADS‐D, SSAS‐D, TICS, BMQ). Inclusion and exclusion criteria were also checked.

On the treatment day, within 28 days of screening, the first of three cold pressor tests (CPTs) was done 1 h before drug administration. Participants received either 15 mg of oxycodone or a placebo, with subsequent CPTs at one and 2 h post‐dose. The placebo capsules were identical in taste, size, and color as the oxycodone capsules. Stress and expectations were measured via a VAS before each CPT. Adverse events were monitored for 7 h, and side effects were recorded at drug administration, 5 h later, and upon next‐day discharge using the GASE questionnaire. All steps were repeated for a second trial phase between 7 and 21 days later, as part of a crossover study designed to simulate a standardized inpatient clinical trial environment.

#### 2.3.4 Study Team Briefing

Before the second cohort (*Arm B*), the study team received a briefing that included an “educational short course” on how to maximize placebo effects. This briefing provided information about the basic mechanisms that initiate a placebo effect, such as subject expectations, context factors, and verbal and non‐verbal communication with the subject. It also covered how to emphasize the positive effects of the drug and how to react and respond to specific questions and comments from the volunteers and certain situations. For the third cohort (*Arm C*), the study team was briefed again, this time with a focus on verbal and non‐verbal communication patterns intended to decrease placebo effects.

A detailed description and a compilation of examples and specific instructions for the study staff can be found in the Supporting Information.

### 2.4 Outcomes and Adverse Events

#### 2.4.1 Primary Outcome

The primary outcome was the change in pain intensity (pain reduction) before and after intake of the study drug assessed by the AUC of the VAS as established by Koltzenburg et al. and Jones et al. [22, 23]. This is a recognized summary metric for continuous CPT pain ratings and has been shown to be sensitive in detecting opioid analgesia [22]. A higher normalized AUC indicates greater individual pain sensitivity: an AUC of 0% denotes a constant VAS pain rating of 0, whereas an AUC of 100% denotes the immediate termination of testing due to pain intolerance, or equivalently, a constant VAS rating of 100 for 120 s. CPT tolerance time, that is, the duration participants withstood the CPT before withdrawing their hand from the water bath, was explored as a secondary outcome. With two subsequent CPTs at one and 2 h post‐dose the pain reduction could be assessed at two different time points. This allowed a differentiated consideration of adverse effects that occurred with time. The primary outcome was analyzed 1 h post‐dose.

#### 2.4.2 Secondary Outcomes

The secondary outcomes of this study included comparing the levels of stress, anxiety, and expectation towards treatment efficacy between the study arms, the incidence of adverse events, and the analysis of the predictability of effects by characteristic traits (anxiety, depression, stress as assessed by the State–Trait Anxiety‐Depression Inventory (STADI), Hospital Anxiety and Depression Scale—German Version (HADS‐D), Generic Assessment of Side Effects (GASE), Somatosensory Amplification Scale (SSAS‐D), and the Trier Inventory for Chronic Stress (TICS)).

### 2.5 Saliva Analysis

Saliva samples were collected by having subjects chew on a synthetic swab from a commercially available collection device (Salivette, Sarstedt, Nümbrecht, Germany) for 1 min. Saliva was then extracted from the swab by centrifugation (1000 × g, 2 min, 4°C) and stored at −80°C until analyzed. Salivary alpha‐amylase activity was measured using an enzymatic assay (Salivary Alpha‐Amylase Assay Kit, Salimetrics Europe, Suffolk, UK) according to the manufacturer's instructions, with a detection limit of 3.28 U/mL. Cortisol was measured in saliva using commercial ELISA assays (IBL International) per the manufacturer's handbook. The detection limits are 0.005 μg/dL and 0.015 μg/dL, respectively. Intra‐assay and inter‐assay variances were 3.2% and 5.3% for alpha‐amylase activity, and 4.8% and 5.9% for cortisol measurements in saliva, respectively.

### 2.6 Statistical Analysis

The study was planned to compare the pain reduction after intake of the study drug as pre‐post treatment differences between the three study arms using a two‐step testing method at a 5% alpha level: first testing for an overall effect, then conducting pairwise comparisons if significant differences emerged.

The primary outcome compared the pain ratings of *Arm A* (unbriefed study team) with those obtained in *Arm B* (optimizing placebo effects) and *Arm C* (attenuating placebo effects). Individual pain intensity was calculated based on the continuous pain rating curves obtained during the CPTs. According to the 2 × 2 cross‐over design in 3 parallel groups with two post‐baseline assessments per period the primary analysis used a mixed linear model. Fixed effects included sequence (oxycodone—placebo or placebo—oxycodone), period (first and second period), study arm (*Arm A, “untrained”; Arm B, “maximize”*; Arm *C, “minimize”*), treatment (oxycodone or placebo), sex, and baseline value (AUC) as a covariate. The model also incorporated a repeated effect of the assessment time within the subjects in a random factor for the given period.

Several sensitivity analyzes were conducted using different models to check the robustness of the results (e.g., using only the first period or a given treatment to compare the effect of the three arms). Additional analyzes explored expectation effects and treatment‐by‐group interactions. Similar mixed models for repeated measures, with adapted assessment time points, were applied to evaluate the time course and effects of the different arms on amylase levels. Results will be presented with effect sizes and 95% confidence intervals, exploring correlations through Pearson and Spearman methods as well as partial coefficients. Secondary endpoints were analyzed using mixed linear models to investigate the influence of various covariates on pain perception under different study conditions.

Sample size was calculated to detect a 10% pre/post difference in VAS scores with 80% power reflecting widely accepted standard parameters for clinical trials. The effect size of 10% change was chosen in line with published recommendations defining a minimally important change of pain intensity [24]. This required 28 subjects per group; 32 were included per group to account for power loss from initial ANOVA steps. The trial adhered to the intention‐to‐treat principle, including all randomized subjects, and used SAS software for analysis.

## 3 Results

### 3.1 Baseline Characteristics of Study Population

**FIGURE 2:**
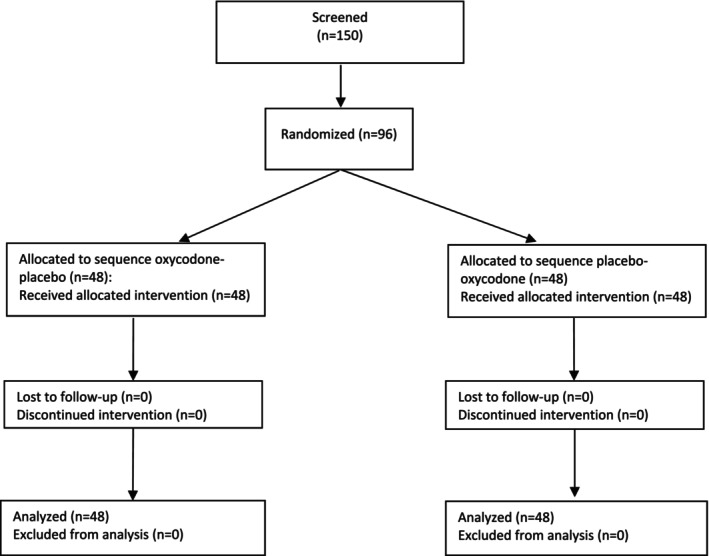
CONSORT‐Flowchart of study participants.

A total of 150 subjects were screened for the study between January 2018 and October 2020. Of these, 96 subjects were randomized to receive the study drug, following either the oxycodone‐placebo sequence or the placebo‐oxycodone sequence, in a crossover design. Each of the three cohorts consisted of 32 subjects. In the first cohort (*Arm A*) there was no briefing of the study team whereas in *Arm B* and *Arm C* the study team was briefed to maximize or minimize the placebo effect, respectively. All subjects completed the study without major deviations from the protocol (Figure 2). Demographics and baseline characteristics were well‐balanced across the three study arms (Table S3). Notably, there were no significant differences in general traits such as depression, anxiety, and stress, assessed using the HADS, STADI, and TICS questionnaires during the screening visit. Furthermore, no significant differences were observed concerning the general attitude towards medication and the tendency to misinterpret somatic sensations, as assessed by the BMQ and SSAS questionnaires.

### 3.2 Primary Outcome: Pain Reduction

**FIGURE 3:**
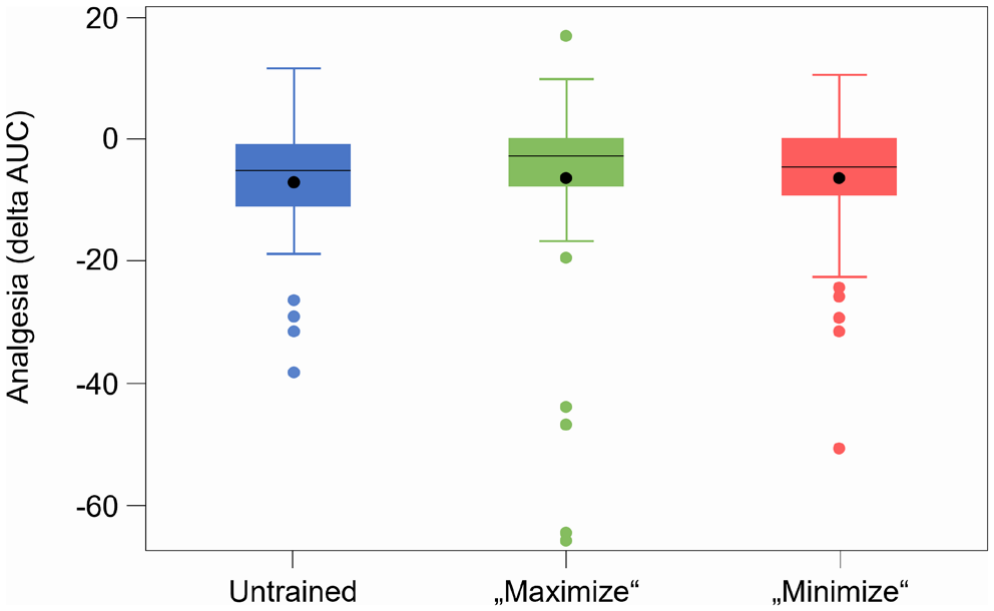
Pain reduction of the study arms. Pain reduction as changes in AUC from pre‐to post‐treatment (delta‐AUC) for the three study arms (A, untrained, B, “maximize”, C, “minimize”) at 1 h post‐dose pooled over both study sequences and treatments (oxycodone, placebo). Within each box, horizontal black lines denote median values; boxes extend from the 25th to the 75th percentile of each group's distribution of values; vertical extending lines denote values within 1.5 interquartile range; dots denote observations outside the 1.5 interquartile range.

In all three cohorts, significant differences in pain perception were noted between oxycodone and placebo (delta‐AUC Arm A: −3.63 vs. −9.64; Arm B: 0.85 vs. −13.06; Arm C: −1.15 vs. −11.09; *p* < 0.001 each; Table S4), validating oxycodone's effectiveness. The true treatment effect varied by 12%–13% from baseline. The magnitude of the placebo effect of the study, best characterized during the first CPT 1 h after placebo administration in period 1, was notably small and varied slightly across the study arms: it was only significant in *Arm A* (−4.0368), very small in *Arm B* (−0.8618) despite efforts to maximize it, and minimally as intended in Arm C (−1.1013). Mixed linear model analysis revealed no significant sex‐related differences. Analysis across the study arms and treatments showed that specific team briefings to alter placebo effects did not significantly impact analgesia in any of the study arms (Figure 3). Baseline pain ratings were consistent across the study arms (*p* = 0.78; Table S5), and there were no significant gender differences in the reduction of pain. Further mixed analyzes indicated no significant influence of baseline pain or psychological variables on post‐treatment pain outcomes.

### 3.3 Subjects Expectation Rating

**FIGURE 4:**
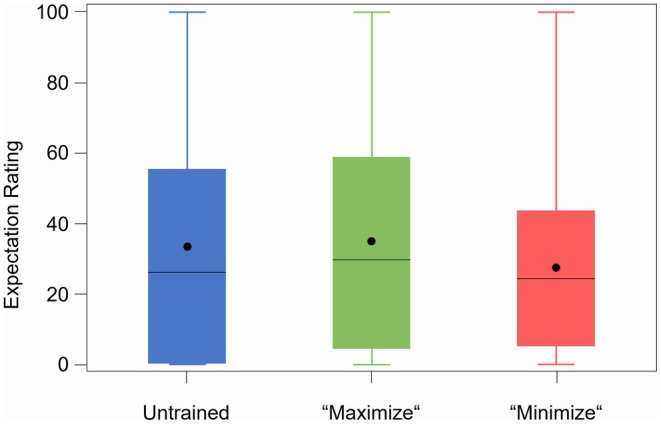
Treatment expectation of the three study arms. Treatment expectation of the three study arms (untrained, “maximize”, “minimize”) averaged from assessments with VAS after intake of the study drug before both CPTs independent from the study treatment applied. Within each box, horizontal black lines denote median values; black dots denote mean values; boxes extend from the 25th to the 75th percentile of each group's distribution of values; vertical extending lines denote values within 1.5 interquartile range; observations outside the 1.5 interquartile range.

As placebo and nocebo effects are based on various mechanisms, with treatment expectations playing a central role, our intervention aimed to influence the treatment expectations of the study participants. However, consistent with the lack of differences in pain reduction in the three study arms described above, there was no significant effect of the different interventions (*Arms A, B* and *C*) on overall treatment expectations regardless of the study treatment applied (oxycodone or placebo) (Figure 4). Only in *Arm C* was the expectation markedly lower under oxycodone (33.7) and similar to that of the placebo group while *Arms A* and B showed very similar expectations under verum (51.4 and 50.9 for Arm A and B, respectively). However, the results indicated at least a tendency towards the intended direction although there was no significant difference owing to the strong variance of the treatment expectations among the three study arms.

**FIGURE 5:**
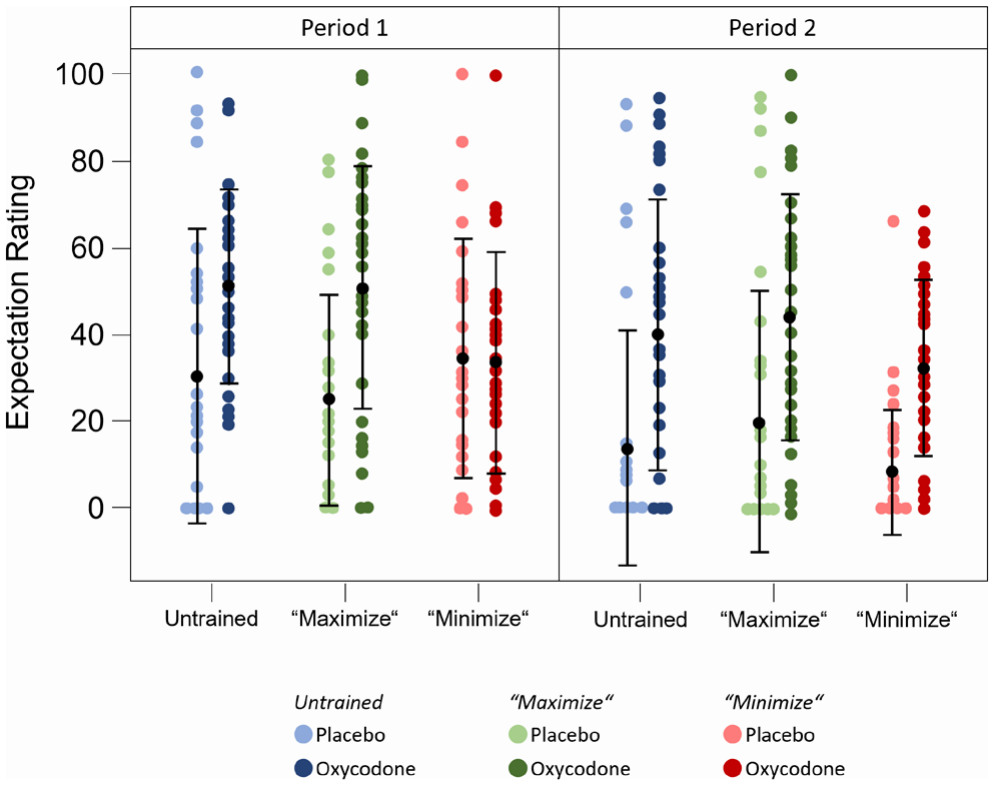
Treatment expectation of the three study arms per period. Treatment expectation of the three study arms (untrained, “maximize”, “minimize”) averaged from assessments with VAS after intake of the study drug before both CPTs separated by study period (1 and 2) and treatment (oxycodone and placebo). Within each column black dots and vertical lines denote means ± SD. SD, standard deviation.

**FIGURE 6:**
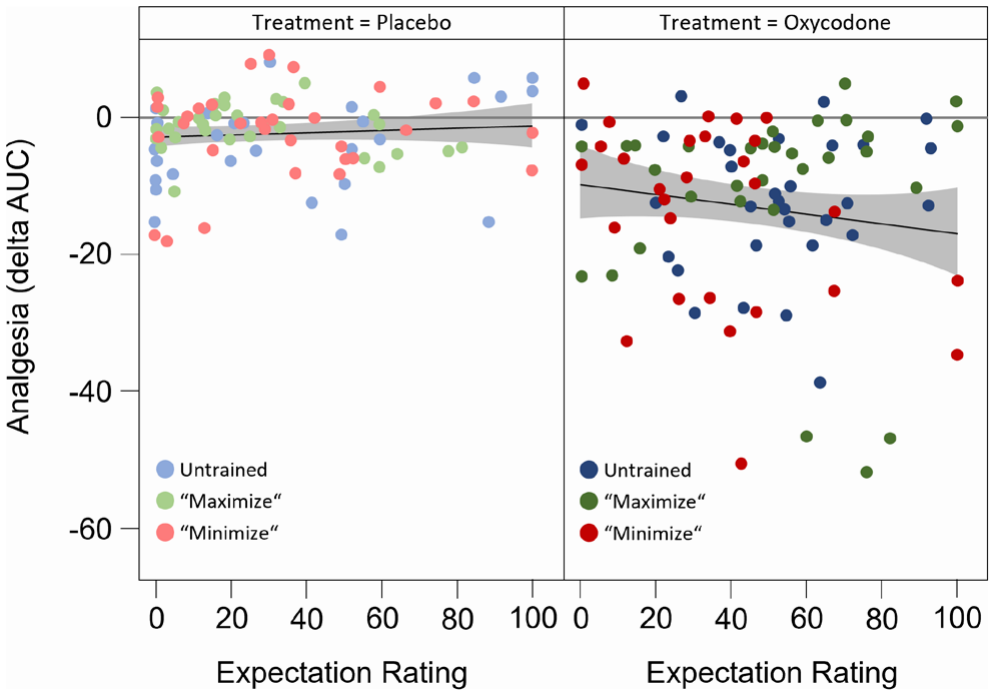
Linear regression of pain ratings and treatment expectation. Linear regression of pain ratings and treatment expectation for the three study arms (untrained, “maximize”, “minimize”) over both study sequences. Pain ratings are illustrated as changes in AUC from pre‐to post‐treatment (delta‐AUC) of the three study arms separated by study period (visit 2 and 5) and study treatment (placebo, left panel; oxycodone, right panel).

When analyzing the different study treatments separately, it was noted that treatment expectations were more pronounced under oxycodone compared to placebo treatment (Arm A: 43.84 vs. 26.58; Arm B: 45.94 vs. 25.42; Arm C: 31.98 vs. 25.35; *p* < 0.01 each) (Figure 5 and Table S6). These findings could be explained by adverse effects in the opioid arm that jeopardize blinding and influence treatment expectations [25]. The unblinding effect of oxycodone was further evidenced by the fact that subjects generally guessed correctly which treatment they had received after completing all study procedures (92.2%). Consequently, the treatment expectations of subjects who received oxycodone during the first period were significantly lower during the second period (data not shown). Furthermore, linear regression analysis of pain ratings with treatment expectation revealed a positive correlation between higher treatment expectation and greater pain reduction. This correlation was observed only after the administration of oxycodone and not after placebo treatment (slope of regression line V slope 0.177) (Figure 6), indicating interactive effects between treatment expectations, pharmacological effects and treatment outcomes.

### 3.4 Salivary Analysis

**FIGURE 7:**
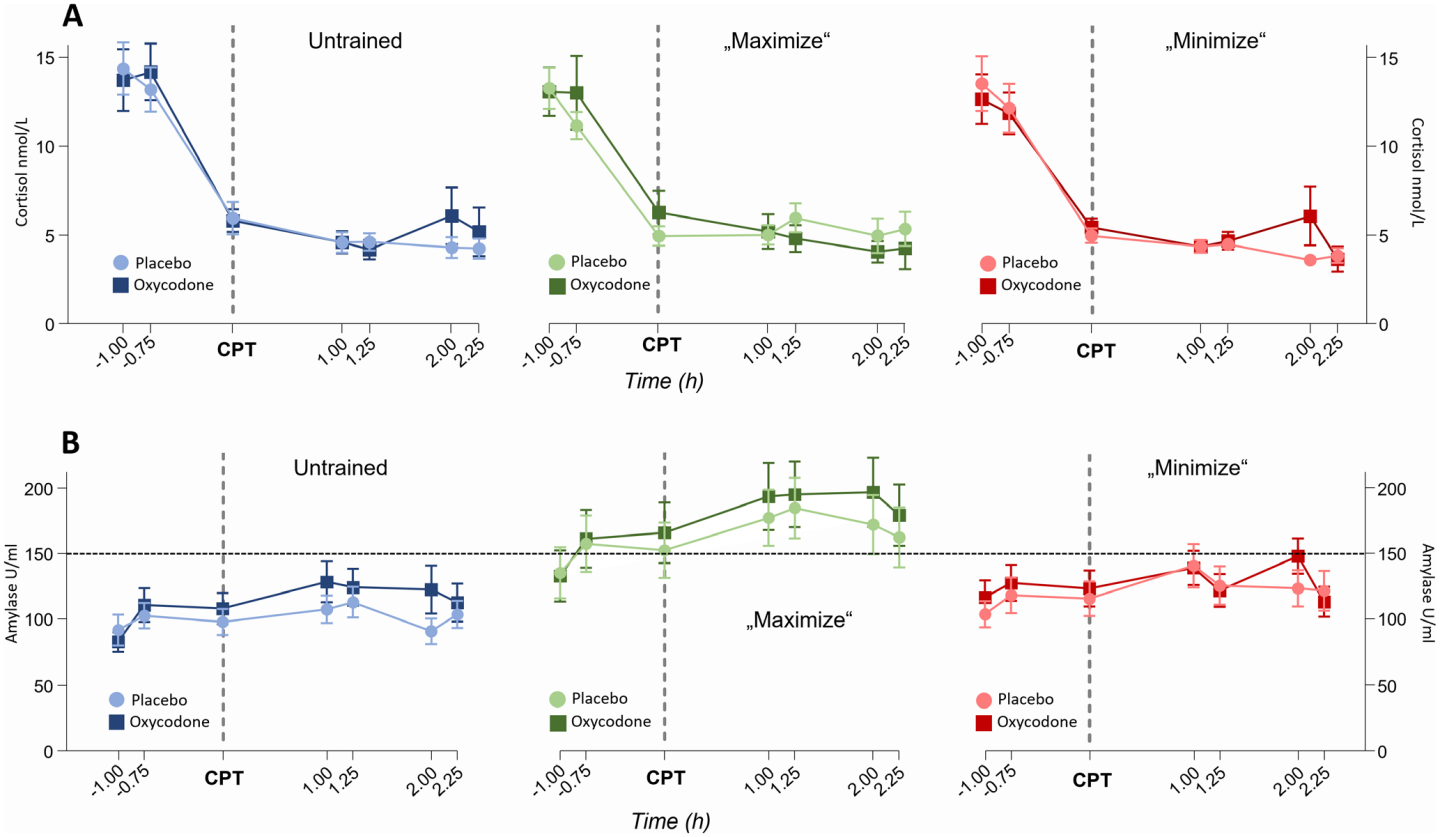
Salivary stress markers. Salivary cortisol levels (A) and alpha‐amylase activity (B) measured at different time points separated (timepoint 0: Intake of the study drug) separated by the three study arms “untrained”, “maximize”, “minimize” (Data are shown as means ± STE). STE, standard error.

Salivary levels of cortisol and alpha‐amylase activity as markers of stress were analyzed to understand whether the different behaviors of the study team had an impact on the psychological stress of the subjects triggered by the pain induction. These are indicators of the activation of the neuroendocrine stress axis, the sympathetic nervous system, and the hypothalamus‐pituitary–adrenal (HPA) axis [26]. Linear regression analysis did not reveal an association between cortisol or alpha‐amylase levels and the reduction of pain perception. Additionally, there were no significant differences in cortisol levels among the three study arms, suggesting that the HPA axis was not significantly affected by the interventions (Figure 7A). However, alpha‐amylase activity was significantly higher during the intervention in *Arm B* compared to *Arm A* and Arm *C* (Figure 7B, *Arm B* vs. *Arm A*: *p* < 0.006, *Arm B* vs. *Arm C*: *p* < 0.042). This increase indicates an activation of the sympathetic‐adrenal‐medullary system in *Arm B* that was likely induced by the intensified interaction of the study team with the participants in this arm.

### 3.5 Adverse Events

Treatment emergent adverse events occurring within 24 h after dosing were generally of mild (83%) or moderate (15%) intensity (Table S8). Naturally, the frequency of adverse events was significantly higher under oxycodone treatment with primarily opiate‐typical side effects (like dizziness, fatigue, nausea) compared to placebo treatment showing rather unspecific symptoms (e. g. headache) indicating unblinding effects of oxycodone. However, there were no relevant differences in the frequency of adverse events between the study arms although there was a slight decrease from *Arm A* to *Arm C* (167, 158, and 148 adverse events). This was also reflected by the patients' rating of side effects (“overall complaints”) using the GASE questionnaire 5 h post dose (*Arm A*: 2.32, *Arm B*: 2.09, *Arm C*: 1.85).

## 4 Discussion

In this randomized, double‐blind, placebo‐controlled crossover study, we investigated whether instructing the study team to modify placebo effects would affect the magnitude of analgesia during placebo and oxycodone treatment. Contrary to our hypothesis, our interventions (*Arm B, “maximize”* and Arm *C, “minimize”* compared to *Arm A, “untrained”*) had only marginal effects on treatment expectations and no significant influence on the analgesic effect of the intervention.

However, our study reveals important associations between treatment expectations and (possibly unblinding) adverse effects which warrant consideration in the design and interpretation of future placebo‐controlled RCTs. Although this study was “negative” with respect to the primary aim, it highlights important aspects for optimizing clinical trial design.

While our education and training of the study team aimed to induce opposing effects on treatment expectations in *Arms B* and *C* (as compared to *A*), the implemented strategy only reduced expectations under verum treatment in *Arm C*. Our results suggest that the complexity and variety of a multi‐day, multi‐visit inpatient study, along with numerous influencing factors, appear to reduce the effectiveness of our targeted interventions by the study staff. Adapting strategies from basic placebo research, typically involving shorter interventions, to real‐world clinical trials clearly requires additional considerations [27]. This is especially true in trials on a specialized phase I unit like in our study. We anticipated that our standardized interventions would better integrate into the structured environment of a phase I trial compared to outpatient phase II or III trials. However, the young, healthy volunteer population showed a high degree of resilience in their treatment expectations and response to placebo effects. This resilience, highlighted by a modest 5.3% placebo effect in *Arm A*—further reduced in *Arms B* and *C*—contrasts with other pain studies reporting placebo effects around 28%. This not only underscores a low baseline expectation during our study but also suggests potential resistance to expectation manipulation supported by experimental evidence that participants with chronic pain conditions display robust placebo effects directly attributable to prior therapeutic experiences [28]. Previous research has shown that simple, short‐term interventions can influence the placebo response [29, 30, 31]. However, maintaining consistent behavioral protocols across multiple visits, including those that span several days, and responding spontaneously to unpredictable situations remain significant challenges for a study team. Given that healthcare providers are not professional actors, strictly adhering to a predetermined script or improvising spontaneously in various situations does not come naturally to them. The effectiveness and credibility of the intervention depend on its authenticity and the individual's acting skills.

Interestingly, our results demonstrate an association between expectation and treatment efficacy. In our experimental approach we used the opioid oxycodone which has been validated in several studies for pain stimulation, demonstrating sufficient efficacy in the cold pressor test model of pain used in this study [18, 19]. This was confirmed by the significant difference in pain reduction between subjects taking oxycodone and those receiving placebo, underscoring the validity of our experimental set‐up. However, oxycodone is known to induce side effects typical of opioids such as nausea, dizziness and fatigue, which can lead to unblinding during a blinded trial. This was supported by the frequency of certain side effects, particularly when compared to placebo and by the fact that subjects generally guessed correctly which treatment they had received after completing all procedures of a study period. Consequently, it was not surprising that treatment expectations were significantly higher under oxycodone compared to placebo.

Our findings were further substantiated by the course of the treatment expectations: during the first period, when participants had no prior experience with the intervention, treatment expectation tended to increase over time under oxycodone (at +2 h lower than at +1 h) but decreased with placebo treatment. Moreover, the treatment expectations of subjects who received oxycodone during the first period were significantly lower during the second period. Finally, we also found that higher treatment expectation was associated with greater treatment efficacy in participants who received oxycodone. Although these findings may seem obvious, they highlight the challenges of conducting blinded, placebo‐controlled trials using a drug with unmasking side effects. If the trial is unblinded in this way, increased treatment expectations might also enhance the treatment effect. This represents a significant confounder leading to the overestimation of specific drug effects compared to placebo. This must be considered when designing, analyzing and interpreting a blinded, placebo‐controlled trial. Our results complement the findings of Schenk et al. [25]. Albeit using a completely different approach, they also showed that side effects can trigger higher treatment expectations and lead to different outcomes.

Even though the varied behavior of the study team did not noticeably affect the expectations and the pain reduction of the participants in general, we observed a significantly increased alpha‐amylase activity during the intervention in *Arms B* compared to *Arms A* and *C*. This indicates an activation of the sympathetic‐adrenal‐medullary system [13] in *Arm B* suggesting that the intervention had an effect on the psychophysiological arousal of the participants. In contrast, this activation was not observed during the neutral behavior of *Arm A* and the indifferent behavior of *Arm C*. Therefore, the concerted efforts by the study team to make the subjects feel comfortable, to emphasize the positive effects of the drug and the increased attention spent, might have paradoxically induced a sense of stress rather than comfort. This is an unexpected and interesting finding as unchanged or even decreased stress levels due to the more sympathetic and empathic team would have been expected. This should be taken into account in the development of future training programs to influence the placebo effect. Interestingly, unlike findings in other studies [32] neither amylase activity nor cortisol levels immediately rose following the pain stimuli from the cold pressor test (data not shown).

It has been shown that training of study participants on report accuracy can increase assay sensitivity [12]. Our data indicate that our brief training of the study team had some influence on the study participants but was not sufficient to minimize and maximize placebo effects in the highly standardized setting of a clinical trial with healthy volunteers as intended. Obviously, the intervention needs to be administered differently, with modified intensity, duration and frequency to improve the effects of the training. This needs to be investigated by future research incorporating biochemical biomarkers, such as plasma proteomics and urinary metabolomics, alongside functional tests to identify predictive signatures of placebo responsiveness and treatment outcome. If the individual behavior of the study team members had a critical influence on trial outcome it would have significant implications for the education and training of study staff as well as for the design of clinical trials to minimize variability of those effects. However, our findings cannot be universally applied to trials involving patients or routine clinical procedures. Patients suffering from symptoms may have a heightened desire for pain relief potentially making it easier to influence their treatment expectations compared to healthy individuals [33]. Therefore, similar approaches should be considered for interventions in patient populations in future studies.

## Author Contributions

M.C., U.B., C.C., M.S. and J.R. designed the study. M.C., M.S., C.C., U.B. and G.H. wrote the manuscript. M.C., M.S., C.C., U.B. and R.N. analyzed the data. M.C., M.S.B. and C.F. performed the research. All authors read and approved the final manuscript.

## Conflicts of Interest

J.R. was employed by Grünenthal GmbH until August 2022. All other authors declared no competing interests for this work.

## Supporting information


**Data S1:** cts70399‐sup‐0001‐SupinfoS1.docx.

## Data Availability

The data supporting the findings of this manuscript will be available from the corresponding author upon reasonable request.
