# Supplementary material for: Modulating Placebo Effects in Clinical Trials: Study Team Briefing to Optimize Drug–Placebo Differences
Source: Clin Transl Sci. 2025 Nov 13;18(11):e70399. doi: 10.1111/cts.70399 (PMC12614085; doi:10.1111/cts.70399)
Supplement: Supplementary file 1 — Data S1: cts70399‐sup‐0001‐SupinfoS1.docx. [file CTS-18-e70399-s001.docx]

**Supplemental Info S1: Detailed information on study coordination and data management**

*Inclusion criteria*

Inclusion criteria applicable for all individuals in the three parts (treatment arms) in this study: males and females aged between 18 and 60 (inclusive) years of age at the screening examination, healthy as determined by a responsible investigator based on a medical evaluation including medical history, physical examination, laboratory tests and 12-lead ECGs. A subject with a clinical abnormality or laboratory parameter(s) outside the reference range which seems irrelevant for the study objectives may be included in consultation with the Principal Investigator. All individuals need to have given their informed consent in writing.

*Exclusion criteria*

The main exclusion criteria include: subjects who are unable to understand the nature, scope, significance and consequences of this clinical study; subjects who are not able to understand and communicate in German as native language; subjects incapable to follow study instructions, to comply with the requirements and restrictions listed in the consent form and to attend and complete all required visits; evidence of acute or ongoing severe infection, current chronic active disease (e. g. cardiac/ pulmonary/ liver/ kidney/ inflammatory/ autoimmune diseases etc.) as assessed by the investigator; known hormonal disease; history of a relevant psychiatric disease; history of chronic pain experience; history of any other relevant disease or condition that, in the opinion of the investigator puts the subject or the study results at unacceptable risk or may interfere with the study procedures and results or with the subject’s participation in this clinical study; relevant concomitant medication (hormones and systematic steroid therapy except contraceptive medication, psychiatric drugs etc.); history of hypersensitivity to the study medication or intolerance of other opioid medication; BMI <18 or >30 kg/m2 (inclusive); history of abuse of medication, drugs or alcohol; preceeding participation in another arm of the study, AUC < 10% in Cold Pressor Test at baseline testing.

**Supplemental Info S2: Detailed information on study coordination and data management**

The planning and execution of the study were supported by the Clinical Study Core Unit (Studienzentrale) of the Study Center Bonn (SZB). This support encompassed statistical analysis, data management, submission to the ethics committee, and clinical trial monitoring. Study data were recorded in source documents and then entered into an electronic case report form (eCRF) provided by REDCap (version 9.5.6) by trained study personnel. REDCap is a secure, web-based software platform designed to support data capture for research studies. It offers audit trails for tracking data manipulation, automated plausibility checks of entered data, and export options for seamless data downloads to common statistical packages. The data were monitored by an experienced clinical study monitor, which included (but was not limited to) checks for the availability of source data, complete source data verification, and confirmation of signatures on all obtained informed consent forms.

The randomization list was generated by the Institute of Medical Biometrics, Informatics and Epidemiology, University of Bonn using the method of permuted blocks separately for the three treatment arms and stratified by gender. Subjects were randomly assigned to one of the blinded treatment sequences of the study medication (Oxycodone – placebo or placebo – Oxycodone) in a 1:1 ratio by sequential allocation of blind-labelled tablet bottles by the study staff. Equal numbers of men and women will be randomized in each treatment arm (A, B and C).

|  |  | **A** (untrained) | **B** („maximize“ placebo effects) | **C** („minimize“ placebo effects) |
| --- | --- | --- | --- | --- |
| n |  | 32 | 32 | 32 |
| Female |  | 16 | 16 | 16 |
| Age |  | 29.63 (10.34) | 32.28 (11.29) | 31.38 (10.85) |
| BMI |  | 23.29 (3.33) | 23.38 (2.72) | 23.46 (2.70) |
| HADS | *anxiety* | 3.22 (3.05) | 2.75 (2.72) | 2.84 (2.32) |
|  | *depression* | 2.00 (2.95) | 0.94 (1.16) | 1.78 (1.86) |
| STADI (trait global) |  | 30.84 (7.51) | 30.13 (5.49) | 30.53 (5.97) |
| TICS |  | 11.63 (8.01) | 9.47 (6.39) | 11.26 (6.30) |
| BMQ | *specific concern* | 8.17 (4.05) | 8.53 (3.55) | 6.91 (1.97) |
|  | *specific necessity* | 8.79 (3.23) | 9.47 (3.88) | 9.18 (4.24) |
| SSAS |  | 22.63 (5.09) | 24.06 (6.20) | 24.25 (5.07) |

**able S3:** Demographics of the subjects of the three cohorts; SD in brackets

BMQ, beliefs about medicines questionnaire; HADS, Hospital Anxiety and Depression; SSAS, Somatosensoric Amplification Scale; STADI, State-Trait-Angst-Depressions-Inventar; TICS, Trierer Inventar zum chronischen Stress; VAS, visual analogue scale;

| **Study arm** | **Treatment** | **Mean (SD)**  **Period 1** | **Median (Period 1)** | **Mean (SD)**  **Period 2** | **Median (Period 2)** |
| --- | --- | --- | --- | --- | --- |
| A („untrained“) | placebo | -4.07 (7.2) | -2.60 | -3.19 (5.62) | -3.55 |
|  | verum | -11.58 (10.21) | -10.7 | -7.71 | -6.15 |
| B („maximize“ placebo effects) | placebo | -0.89 (3.28) | -1.30 | 2.59 (5.89) | 0.75 |
|  | verum | -13.58 (20.33) | -5.95 | -12.53 (17.70) | -7.25 |
| C  („minimize“ placebo effects) | placebo | -1.04 (5.89) | -0.30 | -1.27 (4.52) | -0.60 |
|  | verum | -12.81 (13.97) | -6.50 | -9.56 (8.43) | -8.60 |

**Table S4:** Pain reduction of the study arms as changes in AUC from pre-to post-treatment (delta-AUC) for the three study arms (A, untrained, B, “maximize”, C, “minimize”) at 1 hour post-dose under both study sequences and treatments (oxycodone, placebo)

| **Study arm** | **Treatment** | **Mean (SD)**  **Period 1** | **Median (Period 1)** | **Mean (SD)**  **Period 2** | **Median (Period 2)** |
| --- | --- | --- | --- | --- | --- |
| A („untrained“) | placebo | 73.93 (19.82) | 84.60 | 79.44 (8.11) | 78.05 |
|  | verum | 78.80 (8.69) | 79.20 | 73.99 (21.88) | 77.35 |
| B („maximize“ placebo effects) | placebo | 74.82 (19.14) | 77.70 | 70.95 (25.47) | 81.55 |
|  | verum | 78.46 (11.51) | 81.30 | 74.13 (17.87) | 78.70 |
| C  („minimize“ placebo effects) | placebo | 77.88 (17.83) | 84.3 | 68.34 (20.86) | 70.30 |
|  | verum | 69.62 (18.28) | 74.10 | 76.95 (21.78) | 86.20 |

**Table S5:** Pain ratings (AUC) at baseline

| **Study arm** | **Treatment** | **Mean (SD)**  **Period 1** | **Median (Period 1)** | **Mean (SD)**  **Period 2** | **Median (Period 2)** |
| --- | --- | --- | --- | --- | --- |
| A („untrained“) | placebo | 37.92 (34.54) | 35.60 | 15.24 (28.37) | 0.45 |
|  | verum | 52.41 (21.09) | 52.40 | 35.80 (31.54) | 37.30 |
| B („maximize“ placebo effects) | placebo | 30.29 (26.51) | 24.50 | 20.54 (30.40) | 5.90 |
|  | verum | 53.51 (23.93) | 53.80 | 38.38 (26.52) | 38.45 |
| C  („minimize“ placebo effects) | placebo | 37.42 (28.09) | 35.50 | 11.67 (18.36) | 2.20 |
|  | verum | 34.55 (26.00) | 32.70 | 29.71 (18.13) | 29.90 |

**Table S6:** Treatment expectation of the three study arms (A, untrained, B, “maximize”, C, “minimize”) at 1 hour post-dose under both study sequences and treatments (oxycodone, placebo)

**Supplemental Info S7: Study Team Briefing Protocol**

The brief training sessions generally consisted of five components:

1) A brief interactive lecture on placebo effects, covering mechanisms, influence on drug effects, and the results of experimental and clinical studies. This lecture lasted approximately 45 minutes, with a 30-minute "refresher" for *Arm C*.

2) A presentation of the planned interventions, oriented towards the flowchart of study procedures and key aspects of the intervention's content.

3) Presentations of the planned intervention, including brief video clips showing verbal and non-verbal examples of communication with test subjects by the study team. These videos, recorded at the University Hospital Essen, featured physicians/study personnel demonstrating the positive (*Arm B*) and negative (*Arm C*) variants of communication in typical situations expected during verbal contacts with subjects.

4) Interaction with the study team members and preparation for any additional subject questions that might arise, based on suggestions from the study team.

5) Summaries of the instructions, representative communication and reactions in typical situations were handed out to the study team for repetition during the course of the trial.

The briefing sessions lasted approximately four hours in the first run (*Arm B*) and were shortened to about three hours for the second run (*Arm C*). The sessions were conducted by UB and MS.

1. **Training Setup**

The training program consisted of two training sessions conducted with the study staff, one before Arm B (maximize placebo responses) and one before Arm C (minimize placebo responses).

Both training sessions consisted of a total of 4 components, with a time commitment of approximately four hours for the first session (Arm B) and a shortened version of about three hours for the second session (Arm C, with a short "refresher"). The training sessions were conducted by U. Bingel and M. Schedlowski.

1. **Interactive introductory lecture on placebo:**

Mechanisms, impact on drug effectiveness, and results of experimental and clinical trials
Approx. 90 minutes (approx. 30-minute "refresher" for session Arm C).

1. **Presentation of the specific planned intervention:**

Flowchart and bullet points detailing the content of the intervention.

1. **Presentation of the planned intervention**

Verbal and non-verbal communication with the study participants by the study staff as video clips. These videos were recorded at UK-Essen with professional actors dealing with the positive (Arm B) and negative (Arm C) variations and were shown to the staff being trained, corresponding scripts were handed out.

1. **Team discussion and preparation for potential patient questions**
   Interactive discussion based on suggestions from the study team.

**Dialogue sequences as examples for typical situations during trial conduct:**

**1. Visit 1 (screening visit)**

***A. Significance of clinical trials/drug development"***

- **Opening question**: "Have you ever participated in such studies before?"

**Positive (Arm B)**: Focus on newly developed, promising substances:
"That's great! We are testing new and truly promising substances. It's wonderful that something is being done for pain patients, etc."
"This is a real opportunity and advancement for these patients."

**Negative (Arm C)**: Emphasis on participants as "guinea pigs":
"Well, most studies end up with negative results anyway; it's great that you're helping to ensure no ineffective drugs make it to the market."

***B. Expected efficacy***

- **Opening question**: "What pain relievers have you had experience with?"

**Positive (Arm B)**: "This is completely different, much more significant than something like Ibuprofen. The substance being tested here doesn't just act on tissue but also affects pain processing in the brain."

**Negative (Arm C)**: "You'll be helping to find out whether this drug even works."
"So far, we only know it's safe; but we have no human data yet, and unfortunately, most of these studies fade out, meaning the results never get published."

***C. RCT/Placebo Administration***

- **Opening question**: "Do you know what a placebo is?"

**Positive (Arm B)**:
If Yes: "Exactly, and surprisingly, even placebos often lead to significant pain relief. That's why we need to find out how much better the real painkiller works. You will receive both the placebo and the actual medication."
If No: "Placebos are pills without active ingredients. But surprisingly, even placebos often lead to significant pain relief. That's why we need to find out how much better the real painkiller is. You will receive both the placebo and the actual medication."

**Negative (Arm C)**:
If Yes: "Exactly. In such studies, we use placebos to check whether the new drug is effective. Unfortunately, that often doesn't prove to be the case."
If No: "Placebos are pills without active ingredients. They're used to check whether the new drug is effective. Unfortunately, that often doesn't turn out to be true."

**2. Visit 2: (before breakfast):**

**Morning round**

**Positive (Arm B)**: Study staff communicates empathetically with participants:
"How did you sleep last night? Is everything okay?"
The participant receives reassurance: "We know what we are doing." / "This is routine for us; we do this every day. You couldn’t be in safer hands when taking medication here." (wink)

**Negative (Arm C)**: Study staff communicates distantly and "coldly":
"Good morning, we start in an hour, please be ready."

**3. After breakfast (about 1 hour before medication administration)**

**Reminder**: Emphasizing the possibility of a placebo condition, reminding participants of the setting and randomization. ("You know you could also receive a placebo today.")
Graphical demonstration of placebo vs. active medication.

**Focus on**:
**Positive (Arm B)**: "This placebo condition is crucial for demonstrating the medication's efficacy."

**Negative (Arm C)**: "This placebo condition is crucial because, unfortunately, the drug's effects are often no better than a placebo."

**Short information on side effects**:
Oral reminder and questionnaire.

**Positive (Arm B)**: "It's important not only to see how effective the medication is, but also how well it is tolerated."

**Negative (Arm C)**: "It's important not only to check if it works, but also to monitor side effects and whether it’s tolerable."

**Additional visualization with a diagram of "a pill in the body"**:

**Positive (Arm B)**: "As we know in medicine, no effect comes without side effects. That’s naturally the case with a potent medication like this."

**Negative (Arm C)**: "Side effects often occur because attention is focused on the body, and we become more aware of physical sensations or signals."

"Therefore, even if side effects occur, it doesn't necessarily mean you've received the medication, and certainly doesn't mean it's working."

**4. After Medication/Placebo Administration and Before Cold Pressure Test**

**Short information on using the VAS (Visual Analog Scale)**:
"It’s crucial for the study’s success to use the VAS scale properly, as explained with a diagram."

**Positive (Arm B)**: "We want to know exactly how the medication works; it can vary from patient to patient."
“We would like to measure the level of pain you are currently experiencing. This is a very effective medication, the substance has a much stronger effect than typical painkillers. However, we want to know exactly how strong the effect of the medication is, which can vary from patient to patient. And placebos also have an effect and sometimes better than real painkillers”

**Negative (Arm C)**: "We need to find out if the drug we're testing actually works, and for which patients."

“We would like to measure the level of pain you are currently experiencing. We want to find out whether the medication has any effect at all. Medication often has little better effect than placebos and since there is no active ingredient in them, you can't expect any effect here either.”

**General advice for study staff:**

- do not exaggerate: if the same or different people emphasize the importance of the placebo effect repeatedly in a short period of time, it seems artificial

- don't include the various actions/phrases in a contrived way - it should already fit

- don't just reel off memorized sentences, it has to sound natural

***Specifications for cohort B:***

- all wear uniforms (doctors: coat, study nurses: jacket/white trousers) and name badges to emphasize competence of the role

- introduction with name and “job title”: specialist, nurse, medical student, shake hands (also when saying goodbye at the end)

- friendly/polite behaviour/smile

- empathic communication:

- turn towards the test person/establish eye contact
- “Physical contact” (touching, laying hands on=> Caution: do not appear distenceless)
- Show interest / offer support
- Show understanding (“I can understand that very well”)
- Take your time, listen carefully, do not leave the room in a hurry
- Use emphasizing gestures with body and hands in a meaningful way

- Exude competence / self-confidence

- “this is routine for us”
- “we do that here every day”
- “we have a lot of experience with this”
- “We know what we are doing”
- “there is no safer way to take medication than with us”
- Speak with a firm voice
- If possible, do not make any mistakes/do not let yourself be noticed
- Don't talk about problems/mistakes in front of the subjects
- Keep calm / no hectic
- No ironic remarks

- exude conviction about the study / drug

- focus on / emphasize the good effect of the investigational product:

- “this is a very effective drug”
- “this is a very effective treatment”
- “the treatment is very valuable for pain patients”
- “the substance is much more effective than typical painkillers”
- “the study will help in the development of new pain therapies”

- underline the placebo effect:

- “placebos themselves already work - and sometimes better than real painkillers”
- “This placebo condition is extremely important to show the effectiveness of the medication”

- side effects are only mentioned incidentally / neutrally - i.e. unchanged from cohorts 1 and 3 (possibly suggesting that side effects can also indicate effects)

- we do not go to the subjects more frequently to ask about how they are feeling, but the interaction / communication should be intensified

***Specifications for cohort C:***

- everyone can wear the clothes that suit them, eventually disposable gowns, which create distance without the gown being seen as a “status symbol”.

- rather indifferent behavriour (“we just do our job”)

- distanced and superficial communication:

- Do not show unnecessary interest / offer unnecessary support
- Keep the subjects waiting
- Go to the subjects as rare as possible
- appear rather impatient / rushed (“ where are you?”, “it would be nice if we could continue now”)

- rather radiate uncertainty / indifference

- make a mistakes incidentally / forget something and comment on it (“oh, now I've forgotten that again”)
- Also talk about problems/mistakes in front of the subjects
- “we don't do this that often”
- “I can't tell you that”, “we don't have much experience with that”, “I'd have to look that up again”
- Rather insecure voice
- sometimes become hectic
- cynical comments welcome (“well, that won't help anyway”, “it doesn't really matter”)

- express doubts about the usefulness of the study / drug

- “I don't expect to gain any knowledge from it”
- “Most studies fail anyway”
- “in most studies no effect can be proven”

- emphasize the dubious effect of the investigational drugs

- “whether the drug works remains to be seen”
- “most drugs don't work anyway”
- “I wouldn't expect the drug to have much influence/effect”
- “I guess other painkillers would work better”

- deny the placebo effect:

- “because there's only sugar in placebo, you can't expect any effect here”
- “if you ask me, you could also leave out the placebo arm”

- side effects are only mentioned incidentally / neutrally - i. e. unchanged from cohorts 1 and 2, if necessary mention

- that the medication at least has side effects
- that it is also important to record side effects
- that side effects do not mean that you have received the verum or that the drug is effective

The study team members were instructed to apply various combinations of the provided examples of verbal and non-verbal communication specific for the study arm for all subjects during the first and the second period and to supplement them with their own situationally appropriate formulations with the same communicative intent.

| *Study arm* | *Subject* | *Treatment* | *Event* | *Intensity* | *Related?* | *Outcome* | *Serious* |
| --- | --- | --- | --- | --- | --- | --- | --- |
| A | X001 | Oxycodone | Restlessness | mild | Yes | Completely regressed | No |
| A |  | Oxycodone | Woozy | mild | Yes | Completely regressed | No |
| A |  | Oxycodone | Feeling relaxed | mild | Yes | Completely regressed | No |
| A |  | Oxycodone | Euphoria | mild | Yes | Completely regressed | No |
| A |  | Oxycodone | Tiredness | mild | Yes | Completely regressed | No |
| A | X002 | Oxycodone | Light headedness | mild | Yes | Completely regressed | No |
| A |  | Oxycodone | Nausea | moderate | Yes | Completely regressed | No |
| A |  | Oxycodone | Vomiting | mild | Yes | Completely regressed | No |
| A |  | Placebo | Medical device site haematoma | mild | (unk) | not known | No |
| A | X003 | Oxycodone | Itching | mild | Yes | Completely regressed | No |
| A |  | Oxycodone | Light headedness | mild | Yes | Completely regressed | No |
| A |  | Oxycodone | Feeling of hot flushes | mild | Yes | Completely regressed | No |
| A |  | Oxycodone | Nausea | mild | Yes | Completely regressed | No |
| A |  | Oxycodone | Nausea | mild | Yes | Completely regressed | No |
| A |  | Oxycodone | Hypertension | mild | Yes | Completely regressed | No |
| A | X004 | Placebo | Itchy legs | mild | No | Completely regressed | No |
| A |  | Oxycodone | Head pressure | mild | No | Completely regressed | No |
| A |  | Oxycodone | Deafness | mild | Yes | Completely regressed | No |
| A |  | Oxycodone | Feeling drunk | mild | Yes | Completely regressed | No |
| A |  | Oxycodone | Nausea | mild | Yes | Completely regressed | No |
| A |  | Oxycodone | Itching | mild | Yes | Completely regressed | No |
| A |  | Oxycodone | Foggy feeling in head | mild | Yes | Completely regressed | No |
| A | X005 | Oxycodone | Euphoria | mild | Yes | Completely regressed | No |
| A |  | Oxycodone | Tiredness | mild | Yes | Completely regressed | No |
| A | X006 | Oxycodone | Drowsiness | mild | Yes | Completely regressed | No |
| A |  | Oxycodone | Light headedness | mild | Yes | Completely regressed | No |
| A |  | Oxycodone | Thinking slowed | mild | Yes | Completely regressed | No |
| A |  | Oxycodone | Cotton wool in head | mild | Yes | Completely regressed | No |
| A |  | Oxycodone | Time perception altered | mild | Yes | Completely regressed | No |
| A |  | Oxycodone | Nausea | moderate | Yes | Completely regressed | No |
| A |  | Oxycodone | Pale | moderate | Yes | Completely regressed | No |
| A |  | Oxycodone | Nausea and vomiting | moderate | Yes | Completely regressed | No |
| A |  | Oxycodone | Headache | mild | Yes | Completely regressed | No |
| A |  | Oxycodone | Hiccup | moderate | Yes | Completely regressed | No |
| A | X007 | Oxycodone | Hypertension | mild | Yes | Completely regressed | No |
| A |  | Oxycodone | Bradycardia | mild | Yes | Completely regressed | No |
| A | X008 | Oxycodone | Light headedness | mild | Yes | Improved | No |
| A |  | Oxycodone | Feeling hot | mild | Yes | Improved | No |
| A |  | Oxycodone | Tiredness | mild | Yes | Improved | No |
| A |  | Oxycodone | Nausea | mild | Yes | Completely regressed | No |
| A | X009 | Placebo | Localised muscle pain | mild | No | Completely regressed | No |
| A |  | Oxycodone | Woozy | mild | Yes | Completely regressed | No |
| A |  | Oxycodone | Feeling hot | mild | Yes | Completely regressed | No |
| A |  | Oxycodone | Localised feeling of warmth | mild | Yes | Completely regressed | No |
| A |  | Oxycodone | Vomiting | moderate | Yes | Completely regressed | No |
| A | X010 | Oxycodone | Burning in abdomen | mild | Yes | Completely regressed | No |
| A |  | Oxycodone | Light headedness | mild | Yes | Completely regressed | No |
| A |  | Oxycodone | Disorder sight | mild | Yes | Completely regressed | No |
| A | X011 | Oxycodone | Tiredness | mild | Yes | Completely regressed | No |
| A |  | Oxycodone | Light headedness | mild | Yes | Completely regressed | No |
| A |  | Oxycodone | Bradycardia | mild | Yes | Completely regressed | No |
| A |  | Oxycodone | Itching | mild | No | Completely regressed | No |
| A |  | Oxycodone | Itching | mild | No | Completely regressed | No |
| A |  | Oxycodone | Itching all over | mild | No | Completely regressed | No |
| A |  | Oxycodone | Wound | mild | No | (n.a.) | No |
| A | X012 | Oxycodone | Light headedness | mild | Yes | Completely regressed | No |
| A |  | Oxycodone | Tiredness | mild | Yes | Completely regressed | No |
| A |  | Oxycodone | Euphoria | mild | Yes | Completely regressed | No |
| A |  | Oxycodone | Feeling hot | mild | Yes | Completely regressed | No |
| A |  | Oxycodone | Tired eyes | mild | Yes | Completely regressed | No |
| A |  | Oxycodone | Headache | mild | Yes | Completely regressed | No |
| A |  | Oxycodone | Dry mouth | mild | Yes | Completely regressed | No |
| A |  | Placebo | Headache | mild | Yes | Completely regressed | No |
| A | X013 | Oxycodone | Light headedness | mild | Yes | Completely regressed | No |
| A |  | Oxycodone | Nausea | mild | Yes | Completely regressed | No |
| A |  | Oxycodone | Vomiting | mild | Yes | Completely regressed | No |
| A | X014 | Oxycodone | Tiredness | moderate | Yes | Completely regressed | No |
| A |  | Oxycodone | Twilight state | mild | Yes | Completely regressed | No |
| A |  | Oxycodone | Bradycardia | mild | Yes | Completely regressed | No |
| A |  | Oxycodone | Hunger abnormal | mild | Yes | Completely regressed | No |
| A |  | Oxycodone | Feeling cool | mild | Yes | Completely regressed | No |
| A |  | Oxycodone | Heaviness in limbs | mild | Yes | Completely regressed | No |
| A |  | Oxycodone | Headache | mild | Yes | Completely regressed | No |
| A |  | Oxycodone | Injection site pain | moderate | No | Completely regressed | No |
| A |  | Oxycodone | Vomiting | moderate | Yes | Completely regressed | No |
| A | X015 | Oxycodone | Tiredness | mild | Yes | Completely regressed | No |
| A |  | Oxycodone | Dry mouth | mild | Yes | Completely regressed | No |
| A |  | Oxycodone | Light headedness | moderate | Yes | Completely regressed | No |
| A |  | Oxycodone | Drowsiness | moderate | Yes | Completely regressed | No |
| A |  | Oxycodone | Exhaustion | mild | Yes | Completely regressed | No |
| A |  | Oxycodone | Circulatory instability | mild | Yes | Completely regressed | No |
| A |  | Oxycodone | Avolition | mild | Yes | Completely regressed | No |
| A |  | Oxycodone | Appetite lost | mild | Yes | Completely regressed | No |
| A | X016 | Placebo | Euphoria | mild | Yes | Completely regressed | No |
| A |  | Placebo | Feeling hot | mild | Yes | Completely regressed | No |
| A |  | Placebo | Reading disorder | mild | Yes | Completely regressed | No |
| A |  | Placebo | Tiredness | mild | Yes | Completely regressed | No |
| A |  | Oxycodone | Feeling drunk | mild | Yes | Completely regressed | No |
| A |  | Oxycodone | Fatigue extreme | moderate | Yes | Completely regressed | No |
| A | X021 | Oxycodone | Tiredness | mild | Yes | Completely regressed | No |
| A | X022 | Oxycodone | Bradycardia | moderate | Yes | Completely regressed | No |
| A |  | Oxycodone | Hearing reduced | moderate | Yes | Completely regressed | No |
| A |  | Oxycodone | Localized tingling | moderate | Yes | Completely regressed | No |
| A |  | Oxycodone | Nausea | severe | Yes | Completely regressed | No |
| A |  | Oxycodone | Pale | moderate | Yes | Completely regressed | No |
| A |  | Oxycodone | Cold sweat | moderate | Yes | Completely regressed | No |
| A |  | Oxycodone | Light headedness | moderate | Yes | Completely regressed | No |
| A |  | Oxycodone | Abnormal vision | moderate | Yes | Completely regressed | No |
| A |  | Oxycodone | Nausea | severe | Yes | Completely regressed | No |
| A |  | Oxycodone | Vomiting | severe | Yes | Completely regressed | No |
| A |  | Oxycodone | Pale | mild | Yes | Completely regressed | No |
| A |  | Oxycodone | Nausea | moderate | Yes | Completely regressed | No |
| A |  | Oxycodone | Light headedness | mild | Yes | Completely regressed | No |
| A |  | Oxycodone | Tachycardia | mild | Yes | Completely regressed | No |
| A | X023 | Oxycodone | Woozy | mild | Yes | Improved | No |
| A |  | Oxycodone | Gait instability | mild | Yes | Improved | No |
| A |  | Oxycodone | Tiredness | moderate | Yes | Improved | No |
| A |  | Oxycodone | Nausea | moderate | Yes | Improved | No |
| A |  | Oxycodone | Cold sweat | severe | Yes | Completely regressed | No |
| A |  | Oxycodone | Pale | severe | Yes | Completely regressed | No |
| A |  | Oxycodone | Heart racing | mild | Yes | Completely regressed | No |
| A |  | Oxycodone | Exhaustion | severe | Yes | Completely regressed | No |
| A |  | Oxycodone | Feeling high | moderate | Yes | Completely regressed | No |
| A | X024 | Oxycodone | Hot feeling generalised | mild | Yes | Completely regressed | (n.a.) |
| A |  | Oxycodone | Sweaty hands | mild | Yes | Completely regressed | No |
| A |  | Oxycodone | Woozy | mild | Yes | Completely regressed | No |
| A |  | Oxycodone | Heart racing | mild | Yes | Completely regressed | No |
| A |  | Oxycodone | Feeling drunk | mild | Yes | Completely regressed | No |
| A |  | Oxycodone | Light headedness | moderate | Yes | Completely regressed | No |
| A |  | Oxycodone | Hot feeling generalized | mild | Yes | Completely regressed | No |
| A |  | Oxycodone | Nausea | moderate | Yes | Completely regressed | No |
| A |  | Oxycodone | Tachycardia | mild | Yes | Completely regressed | No |
| A |  | Placebo | Application site itching | moderate | (unk) | not known | No |
| A | X025 | Oxycodone | Tiredness | mild | Yes | Completely regressed | No |
| A |  | Oxycodone | Hypertension | mild | Yes | Completely regressed | No |
| A | X026 | Placebo | Tiredness | mild | Yes | Completely regressed | No |
| A |  | Placebo | Redness in breast | mild | No | Completely regressed | No |
| A |  | Oxycodone | Tiredness | mild | Yes | Completely regressed | No |
| A |  | Oxycodone | Feeling hot | mild | Yes | Completely regressed | No |
| A |  | Oxycodone | Drowsiness | mild | Yes | Completely regressed | No |
| A |  | Oxycodone | Light headedness | mild | Yes | Completely regressed | No |
| A |  | Oxycodone | Dry mouth | mild | Yes | Completely regressed | No |
| A |  | Oxycodone | Redness in breast | mild | No | (n.a.) | No |
| A | X027 | Oxycodone | Tachycardia irregular | mild | Yes | Completely regressed | No |
| A |  | Placebo | Bradycardia | mild | Yes | Completely regressed | No |
| A | X028 | Oxycodone | Foggy feeling in head | mild | Yes | Completely regressed | No |
| A |  | Oxycodone | Dry mouth | mild | Yes | Completely regressed | No |
| A |  | Oxycodone | Tiredness | mild | Yes | Completely regressed | No |
| A |  | Oxycodone | Heartburn | mild | Yes | Completely regressed | No |
| A |  | Oxycodone | Itching | mild | Yes | Completely regressed | No |
| A | X029 | Oxycodone | Headache | mild | Yes | Completely regressed | No |
| A |  | Oxycodone | Light headedness | moderate | Yes | Completely regressed | No |
| A |  | Oxycodone | Inner restlessness | moderate | Yes | Completely regressed | No |
| A |  | Oxycodone | Itching | moderate | Yes | Completely regressed | No |
| A |  | Oxycodone | Tiredness | moderate | Yes | Completely regressed | No |
| A |  | Oxycodone | Sweating increased | mild | Yes | Completely regressed | No |
| A | X030 | Oxycodone | Tiredness | mild | Yes | Improved | No |
| A |  | Oxycodone | Bradycardia | mild | Yes | Completely regressed | No |
| A | X031 | Oxycodone | Drowsiness | mild | Yes | Completely regressed | No |
| A |  | Oxycodone | Light headedness | mild | Yes | Completely regressed | No |
| A |  | Oxycodone | Positional dizziness | mild | Yes | Completely regressed | No |
| A |  | Oxycodone | Tiredness | mild | Yes | Improved | No |
| A | X032 | Oxycodone | Sleepiness | mild | Yes | Completely regressed | No |
| A |  | Oxycodone | Nausea | mild | Yes | Completely regressed | No |
| A |  | Oxycodone | Bradycardia | mild | Yes | Completely regressed | No |
| A |  | Oxycodone | Light headedness | mild | Yes | Completely regressed | No |
| A | X033 | Oxycodone | Light headedness | mild | Yes | Completely regressed | No |
| A |  | Oxycodone | Drowsiness | mild | Yes | Completely regressed | No |
| A |  | Oxycodone | Itching all over | mild | Yes | Completely regressed | No |
| A | X034 | Oxycodone | Light headedness | mild | Yes | Completely regressed | No |
| A |  | Oxycodone | Drowsiness | mild | Yes | Completely regressed | No |
| A |  | Oxycodone | Bradycardia | mild | Yes | Completely regressed | No |
| A | X035 | Placebo | Blood pressure systolic incre | mild | Yes | Completely regressed | No |
| A | X036 | Oxycodone | Light headedness | mild | Yes | Completely regressed | No |
| A |  | Oxycodone | Drowsiness | mild | Yes | Completely regressed | No |
| A |  | Oxycodone | Head pressure | mild | Yes | Completely regressed | No |
| A |  | Placebo | Impaction of intestine | mild | Yes | Completely regressed | No |
| B | X017 | Oxycodone | Tiredness | mild | Yes | Completely regressed | No |
| B |  | Oxycodone | Tiredness | moderate | Yes | Completely regressed | No |
| B |  | Oxycodone | Bradycardia | mild | Yes | Completely regressed | No |
| B |  | Placebo | Tiredness | mild | Yes | Completely regressed | No |
| B |  | Placebo | Hand swelling | mild | Yes | Completely regressed | No |
| B | X018 | Oxycodone | Tiredness | moderate | Yes | Completely regressed | No |
| B |  | Oxycodone | Light headedness | moderate | Yes | Completely regressed | No |
| B |  | Oxycodone | Nausea | moderate | Yes | Completely regressed | No |
| B |  | Oxycodone | Itching | mild | Yes | Completely regressed | No |
| B |  | Oxycodone | Medical device site rash | mild | No | Completely regressed | No |
| B | X019 | Placebo | Bloated feeling | mild | Yes | Completely regressed | No |
| B |  | Oxycodone | Tiredness | moderate | Yes | Completely regressed | No |
| B |  | Oxycodone | Dry mouth | moderate | Yes | Completely regressed | No |
| B |  | Oxycodone | Light headedness | mild | Yes | Not restored | No |
| B |  | Oxycodone | Light headedness | moderate | Yes | Improved | No |
| B |  | Oxycodone | Nausea | mild | Yes | Not restored | No |
| B |  | Oxycodone | Nausea | moderate | Yes | Completely regressed | No |
| B | X020 | Oxycodone | Drowsiness | mild | Yes | Completely regressed | No |
| B |  | Oxycodone | Nausea | mild | Yes | Completely regressed | No |
| B |  | Oxycodone | Bradycardia | mild | Yes | Completely regressed | No |
| B |  | Oxycodone | Skin rash | mild | Yes | Completely regressed | No |
| B |  | Oxycodone | Itching | mild | Yes | Completely regressed | No |
| B |  | Oxycodone | Avolition | moderate | Yes | Completely regressed | No |
| B | X037 | Oxycodone | Perceptual distortion | mild | Yes | Completely regressed | No |
| B |  | Oxycodone | Feeling hot | mild | Yes | Completely regressed | No |
| B |  | Oxycodone | Feeling of relaxation | mild | Yes | Completely regressed | No |
| B |  | Oxycodone | Hypesthesia | mild | Yes | Completely regressed | No |
| B |  | Oxycodone | Feeling high | mild | Yes | Completely regressed | No |
| B |  | Oxycodone | Tiredness | mild | Yes | Completely regressed | No |
| B |  | Placebo | Injection site pain | mild | No | Completely regressed | No |
| B |  | Placebo | Heart rate irregular | mild | Yes | Completely regressed | No |
| B | X038 | Placebo | Blood pressure systolic incre | mild | No | Completely regressed | No |
| B | X039 | Oxycodone | Light headedness | mild | Yes | Completely regressed | No |
| B |  | Oxycodone | Nausea | mild | Yes | Completely regressed | No |
| B | X040 | Oxycodone | Woozy | mild | Yes | Completely regressed | No |
| B |  | Oxycodone | Tiredness | mild | Yes | Completely regressed | No |
| B |  | Oxycodone | Drowsiness | mild | Yes | Completely regressed | No |
| B |  | Oxycodone | Dry mouth | mild | Yes | Completely regressed | No |
| B | X041 | Oxycodone | Headache | mild | Yes | Completely regressed | No |
| B |  | Placebo | Headache | mild | Yes | Completely regressed | No |
| B | X042 | Placebo | Nausea | moderate | Yes | Completely regressed | No |
| B |  | Placebo | Vomiting | mild | Yes | Completely regressed | No |
| B |  | Placebo | Hot feeling generalised | mild | Yes | Completely regressed | No |
| B |  | Placebo | Skin rash | mild | Yes | Completely regressed | No |
| B |  | Oxycodone | Drowsiness | mild | Yes | Completely regressed | No |
| B |  | Oxycodone | Headache | mild | No | Completely regressed | No |
| B | X043 | Oxycodone | Drowsiness | mild | Yes | Completely regressed | No |
| B |  | Oxycodone | Localized tingling | mild | Yes | Completely regressed | No |
| B |  | Oxycodone | Nausea | mild | Yes | Improved | No |
| B |  | Oxycodone | Feeling of hot flushes | mild | Yes | Completely regressed | No |
| B |  | Oxycodone | Period pains | moderate | No | Improved | No |
| B | X044 | Oxycodone | Abnormal physical sensation | mild | Yes | Completely regressed | No |
| B |  | Oxycodone | Drowsiness | mild | Yes | Completely regressed | No |
| B |  | Oxycodone | Hot feeling generalised | mild | Yes | Completely regressed | No |
| B |  | Oxycodone | Time perception altered | mild | Yes | Completely regressed | No |
| B |  | Oxycodone | Dry mouth | mild | Yes | Completely regressed | No |
| B | X045 | Placebo | Bradycardia | mild | Yes | Completely regressed | No |
| B |  | Oxycodone | Hot feeling generalised | mild | Yes | Completely regressed | No |
| B |  | Oxycodone | Drowsiness | mild | Yes | Completely regressed | No |
| B |  | Oxycodone | Feeling relaxed | mild | Yes | Completely regressed | No |
| B |  | Oxycodone | Itching | mild | Yes | Completely regressed | No |
| B |  | Oxycodone | Sweating | mild | Yes | Completely regressed | No |
| B | X046 | Oxycodone | Drowsiness | moderate | Yes | Completely regressed | No |
| B |  | Oxycodone | Light headedness | mild | Yes | Completely regressed | No |
| B |  | Oxycodone | Headache | mild | Yes | Completely regressed | No |
| B |  | Placebo | Interleukin-6 increased | mild | (n.a.) | not known | No |
| B | X048 | Oxycodone | Tiredness | mild | Yes | Completely regressed | No |
| B |  | Oxycodone | Foggy feeling in head | mild | Yes | Completely regressed | No |
| B |  | Oxycodone | Nausea | severe | Yes | Improved | No |
| B |  | Oxycodone | Light headedness | moderate | Yes | Improved | No |
| B |  | Oxycodone | Vomiting | mild | Yes | Completely regressed | No |
| B |  | Placebo | Interleukin-6 increased | mild | Yes | Completely regressed | No |
| B | X049 | Oxycodone | Heaviness of head | moderate | Yes | Completely regressed | No |
| B |  | Oxycodone | Itchy legs | mild | Yes | Completely regressed | No |
| B |  | Oxycodone | Feeling of hot flushes | moderate | Yes | Completely regressed | No |
| B |  | Oxycodone | Dry mouth | mild | Yes | Completely regressed | No |
| B |  | Oxycodone | Light headedness | mild | Yes | Completely regressed | No |
| B |  | Oxycodone | Nausea | moderate | Yes | Completely regressed | No |
| B |  | Oxycodone | Vomiting | mild | Yes | Completely regressed | No |
| B |  | Placebo | Head pressure | moderate | Yes | Improved | No |
| B |  | Placebo | Goose bumps | mild | Yes | Completely regressed | No |
| B |  | Placebo | Interleukin-6 increased | mild | Yes | (n.a.) | Yes |
| B | X050 | Placebo | Procedure site bruising | mild | Yes | Completely regressed | No |
| B |  | Oxycodone | Feeling high | mild | Yes | Completely regressed | No |
| B |  | Oxycodone | Headache | mild | Yes | Completely regressed | No |
| B |  | Oxycodone | Dry mouth | mild | Yes | Completely regressed | No |
| B |  | Oxycodone | Avolition | mild | Yes | Completely regressed | No |
| B |  | Oxycodone | Headache | mild | Yes | Completely regressed | No |
| B | X051 | Oxycodone | Foggy feeling in head | mild | Yes | Completely regressed | No |
| B |  | Oxycodone | Dry mouth | mild | Yes | Completely regressed | No |
| B |  | Oxycodone | Interleukin-6 increased | mild | Yes | not known | No |
| B | X052 | Oxycodone | Nausea | mild | Yes | Completely regressed | No |
| B |  | Oxycodone | Vomiting | mild | Yes | Completely regressed | No |
| B |  | Oxycodone | Tiredness | mild | Yes | Completely regressed | No |
| B | X061 | Placebo | Tiredness | mild | Yes | Completely regressed | No |
| B |  | Placebo | Head pressure | mild | Yes | Completely regressed | No |
| B |  | Placebo | Sweating | mild | Yes | Completely regressed | No |
| B |  | Placebo | Diarrhea | mild | Yes | Completely regressed | No |
| B |  | Oxycodone | Head pressure | mild | Yes | Completely regressed | No |
| B |  | Oxycodone | Feeling of relaxation | mild | Yes | Completely regressed | No |
| B |  | Oxycodone | Localized itching | mild | Yes | Completely regressed | No |
| B | X063 | Oxycodone | Light headedness | mild | Yes | Completely regressed | No |
| B | X064 | Oxycodone | Light headedness | mild | Yes | Completely regressed | No |
| B |  | Oxycodone | Headache | mild | Yes | Completely regressed | No |
| B |  | Placebo | Nausea | mild | Yes | Completely regressed | No |
| B | X065 | Oxycodone | Localized tingling | mild | Yes | Completely regressed | No |
| B |  | Oxycodone | Feeling relaxed | mild | Yes | Completely regressed | No |
| B | X066 | Oxycodone | Headache | mild | Yes | Completely regressed | No |
| B |  | Oxycodone | Feeling of relaxation | mild | Yes | Completely regressed | No |
| B |  | Oxycodone | Talkativeness | mild | Yes | Completely regressed | No |
| B |  | Oxycodone | Light headedness | mild | Yes | Completely regressed | No |
| B |  | Oxycodone | Fuzzy | mild | Yes | Completely regressed | No |
| B |  | Oxycodone | Nausea | moderate | Yes | Completely regressed | No |
| B |  | Oxycodone | Headache | mild | Yes | Completely regressed | No |
| B | X067 | Oxycodone | Light headedness | mild | Yes | Completely regressed | No |
| B |  | Oxycodone | Feeling high | mild | Yes | Completely regressed | No |
| B |  | Oxycodone | Eye accommodation exam | mild | Yes | Completely regressed | No |
| B |  | Oxycodone | Tiredness | mild | Yes | Completely regressed | No |
| B |  | Oxycodone | Localized itching | mild | Yes | Completely regressed | No |
| B | X068 | Placebo | Eye accommodation exam | mild | Yes | Completely regressed | No |
| B |  | Placebo | Cotton wool in head | mild | Yes | Completely regressed | No |
| B |  | Placebo | Euphoria | mild | Yes | Completely regressed | No |
| B |  | Placebo | Tiredness | mild | Yes | Completely regressed | No |
| B |  | Oxycodone | Light headedness | mild | Yes | Completely regressed | No |
| B |  | Oxycodone | Sweating | mild | Yes | Completely regressed | No |
| B |  | Oxycodone | Tiredness | mild | Yes | Completely regressed | No |
| B |  | Oxycodone | Nausea | mild | Yes | Completely regressed | No |
| B | X069 | Placebo | Fumbling | mild | Yes | Completely regressed | No |
| B |  | Placebo | Lassitude | mild | Yes | Completely regressed | No |
| B |  | Placebo | Thought disorder | mild | Yes | Completely regressed | No |
| B |  | Placebo | Increased appetite | mild | Yes | Completely regressed | No |
| B |  | Placebo | Injection site discomfort | mild | No | Completely regressed | No |
| B |  | Placebo | Dry mouth | mild | Yes | Completely regressed | No |
| B |  | Placebo | Restlessness | mild | Yes | Completely regressed | No |
| B |  | Placebo | Orientation disturbed | mild | Yes | Completely regressed | No |
| B |  | Placebo | Abnormal thinking | mild | Yes | Completely regressed | No |
| B |  | Placebo | Hyperaesthesia | mild | Yes | Completely regressed | No |
| B |  | Placebo | Headache | mild | Yes | Completely regressed | No |
| B |  | Placebo | Headache | mild | No | Completely regressed | No |
| B |  | Oxycodone | Perceptual distortion | mild | Yes | Completely regressed | No |
| B |  | Oxycodone | Light headedness | mild | Yes | Completely regressed | No |
| B |  | Oxycodone | Dry mouth | mild | Yes | Completely regressed | No |
| B |  | Oxycodone | Feeling relaxed | mild | Yes | Completely regressed | No |
| B |  | Oxycodone | Headache | moderate | Yes | Completely regressed | No |
| B | X070 | Oxycodone | Feeling high | mild | Yes | Completely regressed | No |
| B |  | Oxycodone | Twitching cervical | mild | Yes | Completely regressed | No |
| B |  | Oxycodone | Sensation of heaviness | mild | Yes | Completely regressed | No |
| B |  | Oxycodone | Food craving | mild | Yes | Completely regressed | No |
| B |  | Oxycodone | Thinking slowed | mild | Yes | Completely regressed | (unk) |
| B |  | Oxycodone | Dry mouth | mild | Yes | Completely regressed | No |
| B |  | Oxycodone | Headache | mild | Yes | Completely regressed | No |
| B |  | Oxycodone | Bradycardia | mild | Yes | Completely regressed | No |
| B | X071 | Oxycodone | Light headedness | mild | Yes | Completely regressed | No |
| B |  | Oxycodone | Tiredness | mild | Yes | Completely regressed | No |
| B |  | Oxycodone | Sweating | mild | Yes | Completely regressed | No |
| B | X072 | Placebo | Headache | mild | Yes | Completely regressed | No |
| B |  | Oxycodone | Headache | mild | Yes | Completely regressed | No |
| B |  | Oxycodone | Bradycardia | mild | Yes | Completely regressed | No |
| C | X053 | Placebo | Tiredness | mild | Yes | Completely regressed | No |
| C |  | Placebo | Headache | mild | Yes | Completely regressed | No |
| C |  | Placebo | Swelling of injection site | mild | No | Improved | No |
| C |  | Oxycodone | Circulatory instability | mild | Yes | Completely regressed | No |
| C |  | Oxycodone | Cotton wool in head | mild | Yes | Completely regressed | No |
| C |  | Oxycodone | Dry mouth | mild | Yes | Completely regressed | No |
| C |  | Oxycodone | Bradycardia | mild | Yes | Completely regressed | No |
| C | X054 | Placebo | Bradycardia | mild | Yes | Completely regressed | No |
| C |  | Oxycodone | Cotton wool in head | mild | Yes | Completely regressed | No |
| C |  | Oxycodone | Hypertension | mild | Yes | Completely regressed | No |
| C |  | Oxycodone | Bradycardia | mild | Yes | Completely regressed | No |
| C | X055 | Oxycodone | Bradycardia | mild | Yes | Completely regressed | No |
| C |  | Oxycodone | Nausea | moderate | Yes | Completely regressed | No |
| C |  | Oxycodone | Tiredness | mild | Yes | Completely regressed | No |
| C |  | Oxycodone | Hypertension | mild | Yes | Completely regressed | No |
| C |  | Oxycodone | Appetite lost | moderate | Yes | Completely regressed | No |
| C | X056 | Oxycodone | Light headedness | mild | Yes | Completely regressed | No |
| C |  | Oxycodone | Tiredness | mild | Yes | Completely regressed | No |
| C |  | Oxycodone | Tiredness | mild | Yes | Completely regressed | No |
| C | X057 | Placebo | Light headedness | moderate | Yes | Completely regressed | No |
| C |  | Placebo | Headache | mild | Yes | Completely regressed | No |
| C |  | Oxycodone | Light headedness | moderate | Yes | Completely regressed | No |
| C |  | Oxycodone | Nausea | moderate | Yes | Completely regressed | No |
| C |  | Oxycodone | Tenseness | moderate | No | Completely regressed | No |
| C |  | Oxycodone | Dry mouth | mild | Yes | Completely regressed | No |
| C |  | Oxycodone | Appetite lost | moderate | Yes | Completely regressed | No |
| C |  | Oxycodone | Avolition | mild | Yes | Completely regressed | No |
| C | X058 | Oxycodone | Attention concentration diffi | mild | Yes | Completely regressed | No |
| C |  | Oxycodone | Feeling hot | mild | Yes | Completely regressed | No |
| C |  | Oxycodone | Dry mouth | mild | Yes | Completely regressed | No |
| C |  | Oxycodone | Drowsiness | mild | Yes | Completely regressed | No |
| C | X059 | Oxycodone | Tiredness | mild | Yes | Completely regressed | No |
| C |  | Oxycodone | Light headedness | moderate | Yes | Completely regressed | No |
| C |  | Oxycodone | Drowsiness | moderate | Yes | Completely regressed | No |
| C | X060 | Oxycodone | Exhaustion | mild | Yes | Improved | No |
| C |  | Oxycodone | Localized itching | mild | Yes | Completely regressed | No |
| C |  | Oxycodone | Feeling of hot flushes | mild | Yes | Completely regressed | No |
| C |  | Oxycodone | Nausea | mild | Yes | Completely regressed | No |
| C |  | Oxycodone | Sweating increased | mild | Yes | Completely regressed | No |
| C | X073 | Oxycodone | Headache | mild | Yes | Completely regressed | No |
| C |  | Oxycodone | Nausea | mild | Yes | Completely regressed | No |
| C |  | Oxycodone | Tingling | mild | Yes | Completely regressed | No |
| C |  | Oxycodone | Gait instability | mild | Yes | Completely regressed | No |
| C |  | Oxycodone | Orientation disturbed | mild | Yes | Completely regressed | No |
| C |  | Oxycodone | Light headedness | mild | Yes | Completely regressed | No |
| C |  | Oxycodone | Generalized anxiety disorder | mild | Yes | Completely regressed | No |
| C |  | Oxycodone | Difficulty in micturition | mild | Yes | Completely regressed | No |
| C |  | Placebo | Bradycardia | mild | Yes | Completely regressed | No |
| C |  | Placebo | Bradycardia | mild | Yes | Completely regressed | No |
| C |  | Placebo | Headache | mild | Yes | Completely regressed | No |
| C | X074 | Oxycodone | Feeling of relaxation | mild | Yes | Completely regressed | No |
| C |  | Oxycodone | Dry mouth | mild | Yes | Completely regressed | No |
| C |  | Oxycodone | Itching | mild | Yes | Completely regressed | No |
| C |  | Oxycodone | Appetite lost | mild | Yes | Completely regressed | No |
| C |  | Oxycodone | Sweating | mild | Yes | Completely regressed | No |
| C | X075 | Oxycodone | Emotional dejection | mild | Yes | Completely regressed | No |
| C |  | Oxycodone | Dry mouth | mild | Yes | Completely regressed | No |
| C |  | Oxycodone | Appetite lost | mild | Yes | Completely regressed | No |
| C | X076 | Oxycodone | Feeling of relaxation | mild | Yes | Completely regressed | No |
| C |  | Oxycodone | Woozy | mild | Yes | Completely regressed | No |
| C |  | Oxycodone | Headache | mild | Yes | Completely regressed | No |
| C | X077 | Oxycodone | Nausea | mild | Yes | Completely regressed | No |
| C |  | Oxycodone | Light headedness | mild | Yes | Completely regressed | No |
| C |  | Placebo | Bradycardia | mild | Yes | Completely regressed | No |
| C | X078 | Placebo | Tiredness | mild | Yes | Improved | No |
| C |  | Oxycodone | Light headedness | mild | Yes | Completely regressed | No |
| C |  | Oxycodone | Tiredness | moderate | Yes | Completely regressed | No |
| C | X079 | Oxycodone | Light headedness | mild | Yes | Completely regressed | No |
| C |  | Oxycodone | Dry mouth | mild | Yes | Completely regressed | No |
| C |  | Oxycodone | Tiredness | mild | Yes | Completely regressed | No |
| C | X081 | Placebo | Feeling hot | mild | Yes | Completely regressed | No |
| C |  | Placebo | Dry mouth | mild | Yes | Completely regressed | No |
| C |  | Oxycodone | Localized tingling | mild | Yes | Completely regressed | No |
| C |  | Oxycodone | Drowsiness | moderate | Yes | Completely regressed | No |
| C |  | Oxycodone | Pressure intraocular increase | mild | Yes | Completely regressed | No |
| C |  | Oxycodone | Tiredness | mild | Yes | Completely regressed | No |
| C |  | Oxycodone | Dry mouth | mild | Yes | Completely regressed | No |
| C | X082 | Oxycodone | Woozy | mild | Yes | Completely regressed | No |
| C |  | Oxycodone | Light headedness | mild | Yes | Completely regressed | No |
| C |  | Oxycodone | Nausea | moderate | Yes | Completely regressed | No |
| C |  | Oxycodone | Vomiting | mild | Yes | Completely regressed | No |
| C |  | Oxycodone | Headache | moderate | Yes | Completely regressed | No |
| C |  | Oxycodone | Medical device site haematoma | mild | No | (unk) | No |
| C | X083 | Placebo | Headache | mild | Yes | Completely regressed | No |
| C |  | Oxycodone | Feeling hot | mild | Yes | Completely regressed | No |
| C |  | Oxycodone | Feeling relaxed | mild | Yes | Completely regressed | No |
| C |  | Oxycodone | Perceptual distortion | mild | Yes | Completely regressed | No |
| C |  | Oxycodone | Tiredness | mild | Yes | Completely regressed | No |
| C |  | Oxycodone | Headache | mild | Yes | Completely regressed | No |
| C |  | Oxycodone | Nausea | mild | Yes | Completely regressed | No |
| C | X084 | Placebo | Haematoma | mild | No | (n.a.) | No |
| C | X085 | Placebo | Exhaustion | mild | Yes | Completely regressed | No |
| C |  | Placebo | Dry mouth | mild | Yes | Completely regressed | No |
| C |  | Placebo | Procedure site bruising | mild | No | (n.a.) | No |
| C |  | Oxycodone | Tiredness | moderate | Yes | Completely regressed | No |
| C |  | Oxycodone | Drowsiness | mild | Yes | Completely regressed | No |
| C |  | Oxycodone | Dry mouth | mild | Yes | Completely regressed | No |
| C |  | Oxycodone | Localized itching | mild | Yes | Completely regressed | No |
| C | X086 | Oxycodone | Light headedness | mild | Yes | Completely regressed | No |
| C | X087 | Oxycodone | Foggy feeling in head | mild | Yes | Completely regressed | No |
| C |  | Oxycodone | Dry mouth | mild | Yes | Completely regressed | No |
| C |  | Oxycodone | Light headedness | moderate | Yes | Completely regressed | No |
| C |  | Oxycodone | Dry eyes | mild | Yes | Completely regressed | No |
| C |  | Oxycodone | Bradycardia | mild | Yes | Completely regressed | No |
| C |  | Oxycodone | Tiredness | moderate | Yes | Completely regressed | No |
| C | X088 | Oxycodone | Light headedness | mild | Yes | Completely regressed | No |
| C |  | Oxycodone | Eye strange sensation of | mild | Yes | Completely regressed | No |
| C |  | Oxycodone | Nausea | mild | Yes | Completely regressed | No |
| C |  | Oxycodone | Light headedness | mild | Yes | Completely regressed | No |
| C |  | Oxycodone | Tremble | mild | Yes | Completely regressed | No |
| C |  | Oxycodone | Inner restlessness | mild | Yes | Completely regressed | No |
| C | X101 | Oxycodone | Woozy | mild | Yes | Completely regressed | No |
| C |  | Oxycodone | Dry mouth | moderate | Yes | Completely regressed | No |
| C |  | Oxycodone | Localised feeling of warmth | mild | Yes | Completely regressed | No |
| C |  | Oxycodone | Tiredness | moderate | Yes | Completely regressed | No |
| C |  | Oxycodone | Feeling of hot flushes | moderate | Yes | Completely regressed | No |
| C |  | Oxycodone | Nausea | moderate | Yes | Completely regressed | No |
| C |  | Oxycodone | Vomiting | mild | Yes | not known | No |
| C | X102 | Oxycodone | Light headedness | mild | Yes | Completely regressed | No |
| C |  | Oxycodone | Sweating | mild | Yes | Completely regressed | No |
| C |  | Oxycodone | Bradycardia | moderate | Yes | Completely regressed | No |
| C |  | Oxycodone | Nausea | mild | Yes | Completely regressed | No |
| C | X103 | Placebo | Tiredness | mild | Yes | Completely regressed | No |
| C |  | Oxycodone | Tiredness | severe | Yes | Completely regressed | No |
| C |  | Oxycodone | Feeling hot | moderate | Yes | Completely regressed | No |
| C |  | Oxycodone | Light headedness | moderate | Yes | Completely regressed | No |
| C | X104 | Oxycodone | Light headedness | mild | Yes | Completely regressed | No |
| C |  | Oxycodone | Sweating | mild | Yes | Completely regressed | No |
| C |  | Oxycodone | Nausea | mild | Yes | Completely regressed | No |
| C |  | Oxycodone | Tremble | mild | Yes | Completely regressed | No |
| C | X105 | Oxycodone | Tiredness | mild | Yes | Completely regressed | No |
| C |  | Oxycodone | Drowsiness | mild | Yes | Completely regressed | No |
| C |  | Placebo | Injection site pain | mild | No | Completely regressed | No |
| C | X106 | Oxycodone | Woozy | mild | Yes | Completely regressed | No |
| C |  | Oxycodone | Dry mouth | mild | Yes | Completely regressed | No |
| C | X107 | Placebo | Headache | mild | Yes | Completely regressed | No |
| C |  | Placebo | Woozy | mild | Yes | Completely regressed | No |
| C |  | Placebo | Bradycardia | mild | Yes | Completely regressed | No |
| C |  | Oxycodone | Tiredness | mild | Yes | Completely regressed | No |
| C |  | Oxycodone | Drowsiness | mild | Yes | Completely regressed | No |
| C |  | Oxycodone | Bradycardia | mild | Yes | Completely regressed | No |
| C | X108 | Oxycodone | Nausea | mild | Yes | Completely regressed | No |
| C |  | Oxycodone | Sweating | mild | Yes | Completely regressed | No |
| C |  | Oxycodone | Light headedness | mild | Yes | Completely regressed | No |
| C |  | Oxycodone | Tiredness | mild | Yes | Completely regressed | No |
| C |  | Oxycodone | Vomiting | mild | Yes | Completely regressed | No |
| C | X109 | Oxycodone | Tiredness | mild | Yes | Completely regressed | No |
| C |  | Placebo | Back pain | mild | No | Completely regressed | No |
|  | | | | | | | |

**Table S8:** Listing of treatment emergent adverse events occurring within 24 hours after dosing by deidentified patients
